# Genome-wide fitness analysis identifies genes required for *in vitro* growth and macrophage infection by African and global epidemic pathovariants of *Salmonella enterica* Enteritidis

**DOI:** 10.1099/mgen.0.001017

**Published:** 2023-05-23

**Authors:** Wai Yee Fong, Rocío Canals, Alexander V. Predeus, Blanca Perez-Sepulveda, Nicolas Wenner, Lizeth Lacharme-Lora, Nicholas Feasey, Paul Wigley, Jay C. D. Hinton

**Affiliations:** ^1^​ Clinical Infection, Microbiology and Immunology, Institute of Infection, Veterinary and Ecological Sciences, University of Liverpool, Liverpool, UK; ^2^​ Department of Clinical Sciences, Liverpool School of Tropical Medicine, Liverpool, UK; ^3^​ Malawi-Liverpool-Wellcome Research Programme, Kamuzu University of Health Sciences, Blantyre, Malawi; ^4^​ Infection Biology and Microbiomes, Institute of Infection, Veterinary and Ecological Sciences, University of Liverpool, Neston, UK; ^†^​ Present address: Department of Laboratory Medicine and Pathology, School of Medicine, University of Washington, Seattle, USA; ^‡^​ Present address: GSK Vaccines Institute for Global Health S.R.L., Siena, Italy; ^§^​ Present address: Wellcome Trust Sanger Institute, Cambridge, UK; ^#^​ Present address: Biozentrum, University of Basel, Basel, Switzerland; ^¶^​ Present address: Bristol Veterinary School,University of Bristol, Langford Campus, UK

**Keywords:** essential genes, fitness, macrophage, *Salmonella *Enteritidis, transposon sequencing

## Abstract

*
Salmonella enterica* Enteritidis is the second most common serovar associated with invasive non-typhoidal *Salmonella* (iNTS) disease in sub-Saharan Africa. Previously, genomic and phylogenetic characterization of *S *. *enterica* Enteritidis isolates from the human bloodstream led to the discovery of the Central/Eastern African clade (CEAC) and West African clade, which were distinct from the gastroenteritis-associated global epidemic clade (GEC). The African *S *. *enterica* Enteritidis clades have unique genetic signatures that include genomic degradation, novel prophage repertoires and multi-drug resistance, but the molecular basis for the enhanced propensity of African *S *. *enterica* Enteritidis to cause bloodstream infection is poorly understood. We used transposon insertion sequencing (TIS) to identify the genetic determinants of the GEC representative strain P125109 and the CEAC representative strain D7795 for growth in three *in vitro* conditions (LB or minimal NonSPI2 and InSPI2 growth media), and for survival and replication in RAW 264.7 murine macrophages. We identified 207 *in vitro*-required genes that were common to both *S *. *enterica* Enteritidis strains and also required by *S *. *enterica* Typhimurium, *S *. *enterica* Typhi and *Escherichia coli *, and 63 genes that were only required by individual *S *. *enterica* Enteritidis strains. Similar types of genes were required by both P125109 and D7795 for optimal growth in particular media. Screening the transposon libraries during macrophage infection identified 177 P125109 and 201 D7795 genes that contribute to bacterial survival and replication in mammalian cells. The majority of these genes have proven roles in *Salmonella* virulence. Our analysis uncovered candidate strain-specific macrophage fitness genes that could encode novel *Salmonella* virulence factors.

## Data Summary

Illumina transposon insertion sequencing (TIS) data have been deposited in the European Nucleotide Archive (ENA) repository (EMBL-EBI) under accession number PRJEB52017. JBrowse genome browsers showing the precise location of transposon insertions across the respective *Salmonella enterica* Enteritidis genomes are available at the URLs https://tinyurl.com/GECP125109 and https://tinyurl.com/CEACD7795. The pipeline for bioinformatic processing and analysis of *S *. *enterica* Enteritidis TIS data is available at the URL https://github.com/apredeus/TRADIS. All Supplementary Material items are available in the Figshare repository at https://doi.org/10.6084/m9.figshare.21720416.v1 [1].

**Impact Statement**

Invasive non-typhoidal *Salmonella* (iNTS) disease is a systemic infection that has a high case fatality rate of 15 % and is responsible for an estimated 66 500 deaths per year in sub-Saharan Africa. The main causative agents are pathovariants of *Salmonella enterica* Typhimurium, known as *S *. *enterica* Typhimurium sequence type 313, and *S *. *enterica* Enteritidis, known as Central/Eastern African and West African *S *. *enterica* Enteritidis. Whilst the African *S *. *enterica* Typhimurium pathovariant has been an active focus of research over the past decade, studies on African *S *. *enterica* Enteritidis have been lacking. We used transposon insertion sequencing (TIS) to identify the genetic requirements of both African and global epidemic *S *. *enterica* Enteritidis to grow *in vitro* and to infect murine macrophages. To our knowledge, this is the first genome-wide functional analysis of African *S *. *enterica* Enteritidis under conditions relevant to infection of a mammalian host. We show that the gene sets required for growth under laboratory conditions and macrophage infection by African and global epidemic *S *. *enterica* Enteritidis were broadly similar, and that the majority of the genes that contribute to survival and replication in macrophages already have proven roles in *Salmonella* virulence. Our analysis identified candidate strain-specific macrophage fitness genes, a potential source of novel *Salmonella* virulence factors.

## Introduction

The majority of human pathogenic *Salmonella* belong to *Salmonella enterica* subspecies 1, including the human-restricted serovars *S *. *enterica* Typhi and *S *. *enterica* Paratyphi (causative agents of typhoid and paratyphoid fever) and host-generalists *S *. *enterica* Typhimurium and *S *. *enterica* Enteritidis commonly associated with gastroenteritis infections. In recent years, non-typhoidal *Salmonella* (NTS) have emerged as the most common cause of community-onset bloodstream infections in sub-Saharan Africa [2, 3]. This invasive non-typhoidal *Salmonella* disease (iNTS) manifests as a febrile systemic illness resembling enteric fever that often lacks gastrointestinal symptoms, and disproportionately affects young children (under the age of 5 years) with co-morbidities such as malnutrition, malaria or human immunodeficiency virus (HIV) infection or HIV-infected adults [4–7]. In 2017, iNTS disease was responsible for 77 500 deaths globally, of which 66 500 deaths occurred in sub-Saharan Africa [7]. The high case fatality rate of iNTS (15 %) [8] makes the disease a major health problem.

Most cases of human iNTS infections across Africa are caused by multi-drug resistant *S *. *enterica* Typhimurium or *S *. *enterica* Enteritidis variants that have characteristic genetic signatures that differ from gastroenteritis-associated *Salmonella *. Approximately one-third of the cases were attributable to *S *. *enterica* Enteritidis, and two-thirds were caused by *S *. *enterica* Typhimurium [2, 3, 9, 10]. African invasive *S *. *enterica* Typhimurium isolates typically belong to the novel MLST (multilocus sequence typing) sequence type 313 (ST313), which is phylogenetically distinct from ST19 that includes most gastroenteritis-associated *S *. *enterica* Typhimurium isolates [9, 11].

A similar theme of phylogeographical differences has been observed in the *S *. *enterica* Enteritidis serovar: two unique clades of invasive *S *. *enterica* Enteritidis have been identified in Africa, designated as the Central/Eastern African clade (CEAC) and West African clade. Both the African clades and the gastroenteritis-associated global epidemic clade (GEC) of *S *. *enterica* Enteritidis belong to ST11 [10]. Overall, the specific genomic signatures of African *Salmonella* isolates that are associated with systemic infection include multi-drug resistance determinants, characteristic prophage repertoires and distinct patterns of genome degradation [9, 10, 12].

*S *. *enterica* Enteritidis GEC strain P125109 and CEAC strain D7795 have been defined as the key representative strains of the respective clades [10]. P125109 was isolated from an outbreak of human food poisoning in the UK in 1988 [13–15], while D7795 was isolated from a blood culture from a Malawian child in 2000 [10]. The genome sequences of P125109 and D7795 were published in 2008 [13] and 2016 [10], respectively, and were recently reannotated and improved with long-read sequencing [16]. The two strains share extensive synteny and collinearity, differing by approximately 1000 SNPs at the whole-genome nucleotide sequence level [10, 16].

To date, genome-wide functional analyses of *S *. *enterica* Enteritidis have focused on gastroenteritis-associated *S *. *enterica* Enteritidis in several *in vitro* conditions, as well as during interaction with human epithelial cells, avian macrophages and during mouse infection [17–20]. Other approaches investigated the role of specific genomic regions such as *Salmonella* pathogenicity islands (SPIs) and regions of difference (RODs) [21–24]. Research on bloodstream-infection-associated African *Salmonella* has been focused on the *S *. *enterica* Typhimurium serovar [25–42]. The Feasey *et al*. [10] and MacKenzie *et al.* [43] studies remain the only two studies that have examined the virulence capabilities of African *S. enterica* Enteritidis, focusing on carbon utilization and biofilm formation *in vitro*, and avian infection *in vivo*. To learn how *S *. *enterica* Enteritidis CEAC strain D7795 causes systemic disease, it was important to determine whether this pathovariant carries genes that have virulence capabilities that were previously unrecognized in GEC strain P125109 or other *Salmonella* serovars in a range of mammalian infection-relevant conditions.

**Fig. 1.**
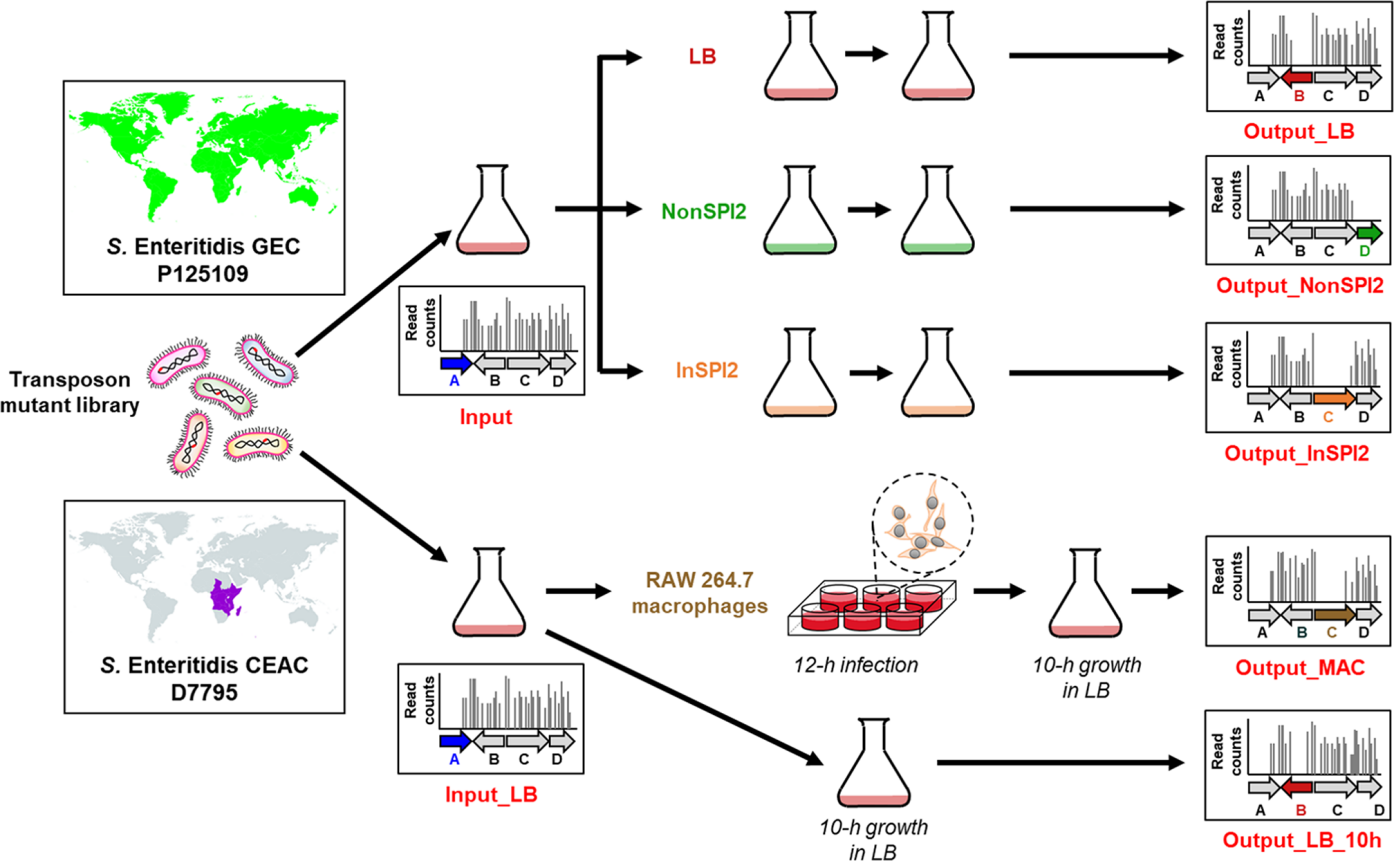
TIS of both *S *. *enterica* Enteritidis GEC strain P125109 and CEAC strain D7795. Schematic representation of the *S. enterica* Enteritidis transposon libraries and growth conditions used in this study. Experimental details are provided in Methods. The genes (*A*, *B*, *C* or *D*) highlighted with colour in the five right-hand panels illustrate how required or fitness genes for a particular environmental condition were identified.

Here, we used transposon insertion sequencing (TIS) to determine the genetic requirements of *S. enterica* Enteritidis GEC strain P125109 and CEAC strain D7795 during growth in four different conditions that represented different aspects of *Salmonella* pathogenesis (Fig. 1). In common with the allied TraDIS and Tn-seq techniques [44], TIS involves random transposon insertional mutagenesis followed by high-throughput sequencing. The relative changes in abundance of each transposon mutant before and after the treatment reflects the contribution of each gene to survival and adaptation to a particular environment. TIS has been successfully used in various bacteria species to define genetic requirements for bacterial viability and fitness during *in vitro* growth and following infection of mammalian hosts and host cells [30, 45–52]. The conditions tested in this study included infection of macrophages, the primary intracellular niche of *Salmonella* during systemic infection [53], and three *in vitro* growth conditions, including a low pH, phosphate-limiting Phosphate-Carbon-Nitrogen (PCN)-based synthetic minimal media named InSPI2, which simulates physiochemical aspects of the intra-macrophage environment, and the corresponding neutral pH, phosphate-abundant NonSPI2 minimal media [54]. Comparisons were made with the nutrient-rich lysogeny broth (LB) media to identify genes required for growth in the respective conditions. We compared the resulting sets of required genes both between GEC strain P125109 and CEAC strain D7795, and against other *Salmonella* serovars used for similar genome-wide functional studies. The genes required for *in vitro* growth and macrophage infection revealed key similarities between the two *S. enterica* Enteritidis strains and extensive correlation with other *Salmonella* serovars. We found a number of genes with the potential of having a previously undescribed strain-specific role in *Salmonella* virulence.

## Methods

### Bacterial strains and growth conditions

Bacterial strains used in this study are detailed in Table S1 (available with the online version of this article). Both the *S. enterica* Enteritidis GEC strain P125109 (NCTC 13349) and the CEAC strain D7795 (NCTC 14676) are available from the National Collection of Type Cultures (NCTC).

The Lennox formulation of LB was routinely used for bacterial cultures, and contained 10 g tryptone l^–1^ (Difco), 5 g yeast extract l^–1^ (Difco) and 5 g NaCl l^–1^ (Sigma). LB agar was prepared by addition of 15 g Bacto agar l^–1^ to LB media prior to autoclaving. InSPI2 (pH 5.8, 0.4 mM inorganic phosphate; P_i_) and NonSPI2 (pH 7.4, 25 mM P_i_) media are PCN-based synthetic minimal media [54, 55]. Terrific broth (TB) (Sigma), a nutrient-rich medium for higher growth of bacteria [56], was prepared according to the manufacturer’s instructions. SOC media contained 20 g tryptone l^–1^, 5 g yeast extract l^–1^, 0.5 g NaCl l^–1^, 2.5 mM KCl, 10 mM MgCl_2_ and 20 mM glucose [57]. When required, the antibiotic kanamycin (Km) was added to a final concentration of 50 µg ml^−1^, tetracycline (Tet) to 25 µg ml^−1^ and gentamicin (Gm) to 20 µg ml^−1^.

### Construction of *S. enterica* Enteritidis transposon mutant libraries

Libraries of transposon insertion mutants were constructed in *S *. *enterica* Enteritidis strains P125109 and D7795 using the EZ-Tn5 <KAN-2> insertion kit (Lucigen) as previously described [30]. Briefly, transposome mixtures were prepared by mixing glycerol, TypeOne restriction inhibitor (Lucigen), EZ-Tn5 <KAN-2> transposon (at 0.1 pmol µl^−1^) and EZ-Tn5 transposase, and electroporated into P125109 or D7795 competent cells. Electro-competent cells were prepared as described previously [58]. Electroporated cells were grown in SOC media for 1 h, before plating on multiple LB agar plates containing 50 µg Km ml^−1^, followed by overnight incubation at 37 °C to select transformants. Following colony counting, the transposon mutants were collected from the plates by adding LB liquid media, and pooling together for growth in LB + 50 µg Km ml^−1^ at 37 °C overnight to generate the transposon mutant library.

### Construction of mutants in *S. enterica* Enteritidis by λ Red recombineering

As negative controls for macrophage infection experiments, *phoPQ* deletion mutants were constructed in P125109 and D7795 using the λ Red recombination method [59], as detailed elsewhere [58]. Briefly, the oligonucleotides phoQ_KO_F and phoPQ_KO_R were used to amplify the Km resistance cassette from the pKD4 plasmid. The PCR product was electroporated into P125109 cells containing the pSIM5-*tet* plasmid [60] and D7795 cells containing the pKD46-*aacC1* plasmid, to replace the *phoPQ* genes. Transformants were selected on LB agar plates containing 25 µg tetracycline ml^−1^ or 20 µg Gm ml^−1^ at 37 °C. The Km resistance cassette from P125109 Δ*phoPQ::aph* and D7795 Δ*phoPQ::aph* were removed by the use of pCP20-*tet* [34] and pCP20-Gm [61] plasmid, respectively. We did not use P22 bacteriophage transduction to move the P125109 Δ*phoPQ::aph* and D7795 Δ*phoPQ::aph* constructions into clean wild-type backgrounds, as P22 did not infect these strains efficiently. To validate the mutants, the P125109 Δ*phoPQ::aph* and D7795 Δ*phoPQ::aph* strains were whole-genome sequenced (MicrobesNG). Bioinformatic comparisons against the P125109 and D7795 genomes [16] confirmed that no unintended mutations had been introduced during the recombineering process (data not shown).

### Passaging the *S. enterica* Enteritidis transposon libraries in LB, NonSPI2 and InSPI2 media

A 1.5 ml aliquot of the P125109 or D7795 transposon library was grown in 25 ml LB + 50 µg Km ml^−1^ in a shaking water bath at 37 °C, 220 r.p.m. for 16 h. Cells harvested from 4×200 µl aliquots of the bacterial overnight culture were stored at −80 °C prior to genomic DNA extraction to give the input sample. Another 1 ml bacterial culture was washed twice with PBS and resuspended in LB, NonSPI2 or InSPI2 media. A 1 : 100 dilution was inoculated into 25 ml LB, NonSPI2 or InSPI2 media (without antibiotic) in 250 ml Erlenmeyer flasks. Cultures were incubated in a shaking water bath at 37 °C, 220 r.p.m. until early stationary phase (ESP) (passage 1). ESP was defined as the moment when the growth of the bacterial culture in the respective growth media first reached a plateau, as measured by OD_600_ readings. The ESP timepoints for P125109 were 7 h (LB), 10 h (NonSPI2) and 10 h (InSPI2), and for D7795 were 6 h (LB), 24 h (NonSPI2) and 24 h (InSPI2).

A total of two passages were performed in each growth medium. For LB passages, 250 µl culture was transferred in each individual passage, following two washes with PBS. For NonSPI2 and InSPI2 passages, to account for the reduced growth that occurred in the minimal medium, a culture volume with OD_600_ equivalent to the LB subculture inoculum was transferred into the subsequent passage to make bacterial numbers as equivalent as possible. For example, if LB passage 1 has an OD_600_ of 2.0 and NonSPI2 passage 1 has an OD_600_ of 1.0, then 500 µl NonSPI2 passage 1 was used to inoculate passage 2. Cells from 4×200 µl aliquots of the second passage of LB (Output_LB), NonSPI2 (Output_NonSPI2) and InSPI2 (Output_InSPI2) were harvested and stored at −80 °C until genomic DNA extraction.

### Infection of RAW 264.7 macrophages with *Salmonella*

For intra-macrophage replication assays with the wild-type and del-*phoPQ* derivatives of *S. enterica* Enteritidis and *S. enterica* Typhimurium strains, 10^6^ RAW 264.7 macrophage cells (ATCC TIB-71) were seeded in each well of 6-well plates (Sarstedt) 24 h prior to infection. Bacterial overnight cultures were inoculated from a single bacterial colony into 25 ml LB, incubated with shaking at 220 r.p.m. and 37 °C for 18 h. Inoculum size was standardized by adjusting the OD_600_ of overnight cultures to OD_600_ 2.0, followed by resuspension in Dulbecco’s modified Eagle's medium (DMEM; Thermo Fisher Scientific) supplemented with MEM non-essential amino acids (NEAA) (Thermo Fisher Scientific; 10 % final concentration) and l-glutamine (Thermo Fisher Scientific; 2 mM final concentration). Prior to all macrophage infection experiments, bacteria were opsonized with 10 % BALB/c mouse serum (Charles River) in 10 volumes of DMEM for 30 min on ice.

The macrophages were infected with *Salmonella* at a m.o.i. of 5–10, and infections were synchronized by 5 min centrifugation at 1000 r.p.m. at room temperature. This was defined as time 0. After 30 min incubation at 37 °C and 5 % CO_2_, cells were washed three times with Dulbecco’s phosphate-buffered saline (DPBS) and incubated with DMEM +10 % FBS (Thermo Fisher Scientific) containing 100 µg Gm ml^−1^ for 1 h to kill extracellular bacteria. For time points beyond 1.5 h post-infection, the cell culture media was replaced with fresh DMEM +10 % FBS containing 10 µg  Gm ml^−1^. Intracellular bacterial numbers were determined by lysis of infected macrophages at 1.5 and 15.5 h post-infection with 1 % Triton X-100 (in DPBS). Serial dilutions of the cell lysates were plated onto LB agar plates (containing antibiotics where necessary) and incubated overnight at 37 °C for bacterial enumeration. Replication fold-change was calculated using the intracellular numbers at 15.5 versus 1.5 h.

For macrophage infection with the *S. enterica* Enteritidis P125109 and D7795 transposon libraries, 10^6^ RAW 264.7 macrophages were seeded in each well of 6-well plates 24 h before infection. A 1.5 ml aliquot of P125109 or D7795 transposon library was grown in 25 ml LB + 50 µg Km ml^−1^ in a shaking water bath at 37 °C, 220 r.p.m. for 16 h, and genomic DNA was isolated from two different biological replicates as input samples (Input_LB_1 and Input_LB_2). OD_600_ of the overnight culture was measured and a bacterial inoculum equivalent to OD_600_ 5.0 was prepared by pelleting and resuspending bacterial cells in appropriate volumes of DMEM supplemented with MEM NEAA and l-glutamine; this equilibration step ensured that sufficient bacterial cells (~1.4×10^7^ cells for P125109 and ~1.5×10^7^ cells for D7795) were used in infection to represent the complexity of the transposon library. Macrophages were infected at an m.o.i. of 5–10 with mouse serum-opsonized bacteria, as described earlier. A total of 18 wells were used in each infection per strain, with six wells set aside for the generation of bacterial counts at 1.5 and 12 h post-infection, and calculation of the fold-change replication of the intracellular bacteria (12 vs 1.5 h).

At 12 h post-infection, macrophages were lysed with 1 % Triton X-100. Macrophage lysates containing intracellular bacteria from 12 wells were pooled into one 15 ml centrifuge tube and centrifuged at 4000 r.p.m. for 5 min. The cell pellet was resuspended in 1 ml LB and transferred to a flask containing 24 ml LB supplemented with 50 µg Km ml^−1^ for 10 h growth at 37 °C, 220 r.p.m. (Output_MAC). To determine the effect of 10 h growth in LB on the transposon library, a fraction of the input library culture was sub-cultured in LB containing 50 µg Km ml^−1^ for 10 h (Output_LB_10 h). Cells were harvested from the output transposon library cultures and stored at −80 °C until genomic DNA extraction.

### DNA manipulation and sequencing

Genomic DNA was purified from all input and output library cultures using the Quick-DNA miniprep plus kit (Zymo Research), following the manufacturer’s instructions. To ensure that sufficient genomic DNA was available for the preparation of Illumina DNA libraries, each DNA sample comprised DNA extracted from 4×200 µl aliquots of the respective bacterial cell samples. DNA concentrations (in ng µl^−1^) were determined using the Qubit dsDNA high sensitivity assay and the NanoDrop 2000 spectrophotometer (Thermo Fisher Scientific).

For Illumina DNA library preparation, 2 µg genomic DNA from each mutant pool was first fragmented to a mean size of 300–350 bp using the BioRuptor@Pico sonication system (15 s on, 90 s off, 9 cycles). Illumina DNA library preparation was performed using NEBNext DNA library prep master mix set for Illumina (New England Biolabs), following the manufacturer’s instructions. Reaction products from each step of library preparation were purified using AMPure XP beads (Beckman Coulter).

To amplify the transposon-flanking regions, transposon-specific forward oligonucleotides (Table S1) were designed such that the first 10 bases of each Read 1 (R1) would be the transposon sequence. A unique 6 base barcode was incorporated into the forward oligonucleotide to allow the pooling of samples for multiplex sequencing in a single lane. A total of 22 cycles of PCR [30, 46] were performed with NEBNext Q5 Hot Start HiFi polymerase using the transposon-specific oligonucleotides and the Illumina reverse primer PE PCR Primer 2.0 for each fragmented DNA sample, following the recommended denaturation, annealing and extension temperatures and durations for NEBNext Q5 Hot Start HiFi polymerase. The resulting DNA was quantified using Qubit dsDNA high sensitivity assay (Thermo Fisher Scientific) and visualized on an Agilent High Sensitivity DNA chip (Agilent Technologies), following the manufacturer’s instructions. Finally, the amplified library was purified with AMPure XP beads and eluted in 30 µl molecular grade H_2_O.

The list of Illumina DNA libraries generated in this study is given in Table S2. For sequencing, the Illumina DNA libraries from P125109 and D7795 were pooled in a ratio corresponding to the difference in estimated library complexity (which was initially defined by the number of transformant colonies) between the two strains. The DNA libraries from RAW 264.7 macrophage infection experiments were pooled in the ratio of 3 : 1 (P125109:D7795). The DNA libraries from *in vitro* passages in LB, NonSPI2, InSPI2 were pooled in the ratio of 2 : 1 (P125109:D7795); the ratio was revised following sequencing of the DNA libraries from macrophage infection experiments, where the actual transposon library densities were revealed to be ~200 000 unique insertions for P125109 and ~100 000 insertions for D7795.

Quality Control assessment of the pooled DNA library and sequencing were performed by the Centre for Genomic Research (CGR), University of Liverpool (UK). Each library pool was size-selected to 250–500 bp, then paired-end sequenced in one lane on an Illumina HiSeq4000 at 2×150 bp (for DNA libraries generated from macrophage infection experiments), generating a total of 340 375 208 paired-end reads. In parallel, for DNA libraries generated from *in vitro* passage experiments, two lanes on an Illumina NovaSeq6000 (SP mode) at 2×150 bp generated a total of 1 048 450 846 paired-end reads (523 450 580 read pairs from the first lane and 525 000 266 read pairs from the second lane). A total of 15 % of the bacteriophage ϕX174 DNA, provided by Illumina as a control, was added to each lane to overcome the low complexity of the bases that followed the barcode in R1 [62]. The sequencing data were processed as described below. Table S2 shows the numbers of sequence reads obtained, the sequenced reads that contained the transposon tag sequence, and the sequence reads that were mapped to the respective *S. enterica* Enteritidis genomes.

### *S. enterica* Enteritidis genome sequences and annotations

The annotated complete long-read-based genome assemblies of *S. enterica* Enteritidis P125109 and D7795 are available in the National Center for Biotechnology Information (NCBI) Assembly database [accession numbers SAMN16552335/GCA_015240635.1 (P125109) and SAMN16552336/GCA_015240855.1 (D7795)] [16]. Orthologues between the *S. enterica* Enteritidis and other *Salmonella* strains presented in this study were identified using the Bacpipe v0.8a pipeline (https://github.com/apredeus/multi-bacpipe). Briefly, Roary v3.13.0 [63] was used to find protein-coding orthologues. Subsequently, nucleotide blast (blastn) was used to identify conserved non-coding small regulatory RNAs (sRNAs), genes encoding small proteins and pseudogenes, by comparison with a reference list of small protein and non-coding sRNA genes identified in *S. enterica* Typhimurium ST19 and ST313 (available at https://github.com/apredeus/multi-bacpipe/blob/master/reference/St_ncNRA_sORF.fa), using the ‘-r’ option in ‘prepare_bacterial_reference’ command. Final validation of the annotations of the P125109 and D7795 genomes was achieved manually by comparing the annotations to the locus tags in the published P125109 annotation [13]. Cluster of Orthologous Genes (COG) categories were assigned with eggNOG-mapper v2 [64, 65] using the default parameters.

### Sequence analyses of the *S. enterica* Enteritidis transposon library

Bioinformatic processing and analysis of *S. enterica* Enteritidis transposon insertion data followed the general strategy described elsewhere [30, 46, 62], with modifications. The code and full description of the analysis pipeline are available at https://github.com/apredeus/TRADIS.

Raw sequencing data was demultiplexed using cutadapt v2.6 [66]. First, a barcode sequence fasta file that included one sequence per sample was compiled, after which cutadapt was run with options ‘cutadapt -O 34 g file:barcodes.fa --discard-untrimmed’. This generated a set of two paired-end fastq files for each sample. The reads were then aligned to the reference genome using bwa v0.7.17-r1188 (using bwa mem algorithm). Aligned BAM files were sorted and indexed using SAMtools v1.9 [67].

For further processing, two GFF annotation files were generated for each bacterial strain used in the experiments. One file was used for deduplicated read counting and was obtained from a general annotation file by changing the type of each feature that had a locus tag (ID) into ‘gene’. The second GFF file was generated the same way, with additional change to the annotated feature size: the last 10 % of each annotated gene was removed. This annotation file was used in essentiality analysis, since previous reports [62, 68] showed that insertions or deletions in the last 10 % of the gene are much less likely to cause a complete loss of function.

Following these steps, a series of additionally processed alignment (BAM) files was created. First, picard MarkDuplicates v2.21.2 was used to remove PCR and optical duplicates from the aligned reads. The resulting files were filtered using *cigar_filter.pl* to select only the reads that align exactly at the start of R1 without softclipping. The resulting filtered BAM files were converted into 1 nt single-end BAM files using *make_1nt.pl* script to avoid counting reads that spanned into the nearby genes. These alignment files were used for quantification of deduplicated reads and used in DESeq2 analysis. In parallel, the filtered BAM files were also converted into 1 nt unique insertion BAM files using *make_1nt_uniq.pl* script; these files were used for essentiality analysis.

The resulting 1 nt BAM files were quantified using featureCounts v1.6.4 [69] with '-M -O --fraction -t gene -g ID -s 0' options for DESeq2 analysis, and '-M -O -t gene -g ID -s 0' options for essentiality analysis. This was done to account for multimapping reads: if only uniquely mapping reads were considered, transposable and other repetitive elements looked falsely essential. Indeed, if multimapping reads were discarded, genes that have multiple copies (such as transposons) appear to have a zero transposon insertion rate, which in turn leads us to the false conclusion of their essentiality.

### Essentiality analysis

Essentiality analysis was done using the unique insertion counts. For consistency with our DESeq2-based fitness analysis, we have re-implemented the essentiality analysis functions of Bio-TraDIS [62], specifically R functions *make_ess_table* and *calculate_essentiality*. Briefly, the unique insertion counts were converted into an insertion index (that is, insertion sites divided by gene length) [46], which followed the expected bimodal distribution. The distribution histogram was used to fit two functions: exponential function for very low insertion indices corresponding to the required genes, and gamma function for high insertion indices of the dispensable genes. Using the obtained fits, the genes were classified as follows: if log-likelihood of the exponent to gamma distribution was 2 or higher, the gene was deemed ‘required’; if the ratio was below −2, it was deemed ‘not required’; genes with intermediate insertion indices were reported as ‘ambiguous’. Due to the relatively low insertion densities in our libraries, essentiality calls were not assigned for features shorter than 200 nt.

Data from the TIS-based study on *S. enterica* Typhimurium ST313 D23580 [30] were used as a comparator to identify common *Salmonella* genes that were required for growth under laboratory conditions or during macrophage infection, and differences that might reflect unique requirements for each serovar or the pathogenic niche inhabited by these bacteria. Due to the substantial differences in the mean number of unique insertions present in the transposon library for *S. enterica* Enteritidis GEC P125109, CEAC D7795 and *S. enterica* Typhimurium D23580, we used both deduplicated read counts and essentiality calls to identify robust differences.

Deduplicated read counts for LB input, LB output and macrophage output for the three strains were log_2_-transformed and quantile-normalized. These values were then used to calculate a log_2_ fold-change between the libraries that was evaluated and found to follow an approximately normal distribution (data not shown). Thus, we have selected the genes that were significantly different in all three conditions in the particular strains according to *t*-test (*P* value ≤0.05), and also had differing essentiality calls. This approach allowed us to identify 63 genes that satisfied both conditions. The resulting genes were visualized using the Phantasus gene expression analysis tool (http://genome.ifmo.ru/phantasus-dev/). K-means clustering of rows allowed us to identify five distinct groups of genes, according to the strain they were required in (Fig. S1).

### DESeq2-based fitness analysis

Analysis of differential fitness was performed in R v4.0.2, using DEseq2 v1.28.1 [70] and a simple study design (~ Condition) with default settings. Deduplicated raw read counts were used as expression values. Each condition was represented by at least two biological replicates. Results are shown in log_2_ fold-change. A cut-off of 2-fold-change and *P* value <0.05 was applied.

### Statistical analyses of intra-macrophage replication experiments

Statistical analyses were performed with GraphPad Prism 7 (version 7.04). Ordinary one-way ANOVA and the Bonferroni’s multiple comparison test were used to determine differences in intra-macrophage replication levels between different *Salmonella* strains. A *P* value of less than 0.05 was considered to be statistically significant.

## Results and Discussion

### Characterization of the *S. enterica* Enteritidis P125109 and D7795 transposon libraries

Transposon insertion libraries were constructed in the *S. enterica* Enteritidis GEC representative strain P125109 and CEAC representative strain D7795. Each pool of transposon mutants was grown in LB (Input) and passaged two times successively at 37 °C in three different growth media: a nutrient-rich medium, LB (Output_LB), an acidic (pH 5.8) phosphate-limiting PCN-based minimal media that reflects aspects of the intra-macrophage environment and induces SPI-2 gene expression [54], designated InSPI2 (Output_InSPI2), and a neutral pH PCN-based minimal media that does not induce SPI-2 gene expression [54], designated NonSPI2 (Output_NonSPI2) [55] (Fig. 1).

Following isolation of genomic DNA from mutant pools and Illumina sequencing, the input libraries identified 246 743 unique transposon insertion sites in P125109 and 195 646 unique insertions in D7795, a mean of one insertion per 19 nt in the P125109 genome and one insertion per 24 nt in the D7795 genome. The complete data sets are available for visualization in two online JBrowse genome browsers for the respective strains: https://tinyurl.com/GECP125109 and https://tinyurl.com/CEACD7795. The browsers show the transposon insertion profiles for the chromosome and plasmid of *S. enterica* Enteritidis (pSENV in P125109; pSEN-BT and pRGI00316 in D7795). To yield maximum biological insight from the transposon mutagenesis experiment, we employed our recent re-annotation of the coding genes and non-coding sRNA genes of P125109 and D7795 [16], which was derived from a comparative genomic approach that identified all the annotated genes of *S. enterica* Typhimurium ST19 [71] and *S. enterica* Typhimurium ST313 [31] that were carried by the two *S. enterica* Enteritidis strains (see Methods).

An insertion index was calculated for each gene by dividing the number of unique insertions for any given gene by gene length; the data were used for essentiality analyses (see Methods), classifying genes as required, not required and ambiguous. Required genes in this study included genes essential for bacterial viability (i.e. genes that when disrupted lead to irreversible growth arrest or cell death), and genes that contribute strongly to fitness in a particular environmental condition [30, 72]. The role of genes shorter than 200 nt in length could not be defined robustly and the genes were designated as ‘short’. The number of reads, transposon insertion sites, insertion index and the essentiality calls per gene for all conditions tested are summarized in Table S3.

We considered one potential caveat of global transposon mutagenesis, namely that transposon orientation might have polar effects on the expression of adjacent genes. Such an issue could theoretically lead to the incorrect assignment of fitness phenotypes to certain genes. Previous work by our group [30] and others [73] has provided evidence that, in the majority of insertions, transposon orientation did not impact upon mutant fitness.

### Identification of *S. enterica* Enteritidis P125109 and D7795 genes required for *in vitro* growth

**Fig. 2.**
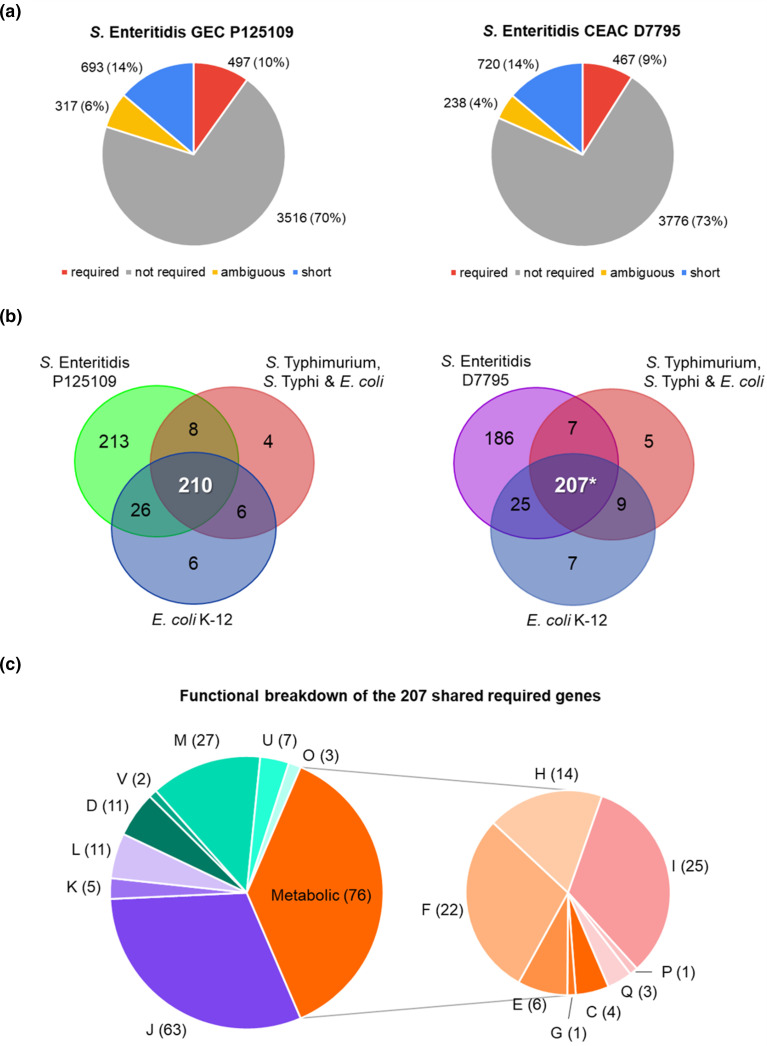
*
S *. *enterica* Enteritidis GEC P125109 and CEAC D7795 genes required for *in vitro* growth. (a) Distribution of *S. enterica* Enteritidis GEC P125109 (left panel) and CEAC D7795 (right panel) genes into required, not required, ambiguous and short (gene length <200 nt) categories. Full gene lists are presented in Table S3. (b) Comparison of required genes identified in GEC P125109 and CEAC D7795 with published gene essentiality studies for *S. enterica* Typhimurium [45], *S. enterica* Typhi [45] and *E. coli* [45, 68]. Only genes that shared an orthologue in P125109 or D7795 were used in the comparison. The asterisk (*) indicates that all the 207 D7795 genes were included in the 210 required P125109 genes (Table S4). Venn diagrams were generated using http://bioinformatics.psb.ugent.be/webtools/Venn/. (c) COG functional breakdown of the 207 required genes shared between P125109, D7795, *S. enterica* Typhimurium, *S. enterica* Typhi and *E. coli *. The COG categories are as follows: J, translation, ribosomal structure and biogenesis; K, transcription; L, replication, recombination and repair; D, cell cycle control, cell division, chromosome partitioning; V, defence mechanisms; M, cell wall/membrane/envelope biogenesis; U, intracellular trafficking, secretion and vesicular transport; O, posttranslational modification, protein turnover, chaperones; C, energy production and conversion; G, carbohydrate transport and metabolism; E, amino acid transport and metabolism; F, nucleotide transport and metabolism; H, coenzyme transport and metabolism, I, lipid transport and metabolism; P, inorganic ion transport and metabolism; Q, secondary metabolites biosynthesis, transport and catabolism.

Essentiality analysis of the *S. enterica* Enteritidis GEC strain P125109 input library identified 497 required genes, 3516 dispensable genes and 317 ambiguous genes, with 693 genes being classified as short (Fig. 2a). Of the 497 required genes (Table S3), 492 genes were chromosomally located and 5 genes (*traJ*, *samA*, *samB*, *SEN_p0037* and *SEN_p0021*) were carried by the 59 kb pSENV virulence plasmid [16]. To provide a functional context for the required genes, we used eggNOG-mapper v2 to assign COG functional categories (Table S3). The majority of the required genes were involved in translation (J, 17 %) and cell wall biogenesis (M, 5 %), followed by an approximately equal distribution between the categories of transcription (K, 3 %), replication (L, 3 %), energy production (C, 3 %) and various metabolic processes (E, F, H and I). Ninety-six genes (16 %) were classified in the ‘poorly characterized’ category, including 28 genes belonging to RODs [13], prophage regions and SPIs. Eleven SPI-associated genes were required by P125109 for *in vitro* growth, including several *ssa* and *ttr* genes in SPI-2, *SEN0277* in SPI-6 and *SEN4250* in SPI-10.

Essentiality analysis of the *S. enterica* Enteritidis CEAC strain D7795 input library identified 467 required genes, 3776 dispensable genes, 238 ambiguous genes and 720 short genes (Fig. 2a). Of the 467 required genes (Table S3), 465 genes were encoded in the chromosome and two genes (*samA* and *SEN_p0046*) were located on the 116 kb virulence plasmid pSEN-BT; no required genes were carried by the smaller 4.9 kb plasmid pRGI00316. COG categories were assigned to the D7795 reference genome as described earlier. As seen for P125109, the majority of the D7795 required genes are involved in translation (J, 18 %), cell wall biogenesis (M, 9 %) and nucleotide metabolism (F, 7 %). Nine required genes were associated with pathogenicity islands, namely four SPI-2 genes (*ssaT*, *ssaH*, *ttrAB*), two SPI-6 genes (*SEN2077* and *SEN2078*), two SPI-10 genes (*SEN4248* and *SEN4250*) and one SPI-14 gene (*SEN0801*).

Several genes identified as required for *in vitro* growth in both *S. enterica* Enteritidis strains are counter-intuitive, e.g. the SPI and plasmid-encoded *tra* genes. While previous TIS studies in *S. enterica* Typhimurium also reported a requirement of certain SPI genes for growth in laboratory conditions [30, 45], the generation of viable null *S. enterica* Typhimurium and *S. enterica* Enteritidis mutants in various SPI-located genes indicates that these are not essential genes in both *Salmonella* serovars [30, 45, 74–78]. We hypothesize that the low number of transposon insertions in the required SPI genes reflects the association of the histone-like nucleoid structuring (H-NS) protein with the SPIs. The binding of H-NS to chromosomal regions has been reported to occlude access to transposons [79]. Horizontally acquired genes such as SPIs are characterized by their lower G+C content. In many bacterial species, H-NS preferentially binds to such AT-rich regions in the genome, a mechanism that silences expression of foreign genes [80–82]. The binding of H-NS to SPI regions has been well documented in *S. enterica* Typhimurium [80, 81]. To confirm our hypothesis, in future, the same chromatin-immunoprecipitation approach should be used to determine the binding pattern of H-NS across the *S. enterica* Enteritidis genome.

### Total of 207 required genes shared between *S. enterica* Enteritidis P125109 and D7795, as well as *S. enterica* Typhimurium, *S. enterica* Typhi and *Escherichia coli*

To put our essentiality analysis into context, we drew upon other studies that used similar TIS approaches. The required genes of *S. enterica* Enteritidis GEC P125109 and CEAC D7795 were compared with the genetic requirements of *Salmonella* serovars Typhimurium and Typhi, and *E. coli *. We used two required gene sets: the first is a list of 228 essential genes shared between *S *. *enterica* Typhimurium, *S *. *enterica* Typhi and *E. coli* [45], and the second is a list of 248 essential genes identified in *E. coli* K-12 [68]. We found that a total of 207 genes were required across *S. enterica* Enteritidis, *S. enterica* Typhimurium, *S. enterica* Typhi and *E. coli* (Fig. 2b); a recent comparative TraDIS study also identified a similar number of 201 universally essential genes in *Enterobacteriaceae* [83]. The 207 required genes mainly encode the basic cellular machinery (e.g. DNA replication and protein translation) and pathways vital for the growth of the bacteria, including cell wall biogenesis and cell division (Fig. 2c, Table S4). Of the six and nine genes identified as required in *S *. *enterica* Typhimurium, *S *. *enterica* Typhi and *E. coli* but not in P125109 or D7795, respectively, most were designated as short genes (gene length less than 200 nt) in either *S. enterica* Enteritidis strain and, therefore, had not been assigned an essentiality call. Such genes included *infA*, encoding translation initiation factor IF-1; *csrA*, encoding a post-transcriptional regulator that regulates metabolism important for establishing infection in the intestine [84]; and *rpmD*, *rpmC* and *rpmH*, encoding 50S ribosomal proteins. The *ispB* gene was the only one of these genes identified as not required in D7795. Overall, our observations were in agreement with other reports: required genes are conserved among bacterial strains within the same species or between different species [30, 45, 47, 49, 85].

### Sixty-three orthologous genes that are only required by *S.* enterica Enteritidis or *S *. *enterica* Typhimurium D23580

The Venn comparisons in Fig. 2 suggest there are approximately 200 genes that are only required by *S. enterica* Enteritidis, but dispensable in *S. enterica* Typhimurium, *S. enterica* Typhi and *E. coli *. However, the differences that arise from such direct comparisons involving different TIS-based studies can be challenging to interpret, in part due to the differences in experimental protocols, transposon library profiles and bioinformatic processing pipelines used by different laboratories. Consequently, we made use of data from our recently published TIS-based analysis of genetic requirements of *S. enterica* Typhimurium ST313 D23580 for survival and growth both *in vitro* and during macrophage infection [30] to identify differences in the genetic requirements with *S. enterica* Enteritidis P125109 and D7795. To address the issue of the different insertion densities of the two *S. enterica* Enteritidis transposon libraries (~200 000 each) and the *S. enterica* Typhimurium ST313 D23580 library (at least ~500 000), a customized pipeline (see Methods) was used to compare the three strains and to perform essentiality analysis. Both essentiality calls (using insertion indices) and changes in abundance of read counts in three conditions (LB input, LB output and macrophage output) were used in the inter-strain essentiality analysis.

A total of 63 orthologous genes were identified as differentially required by *S. enterica* Enteritidis P125109 and D7795 and *S. enterica* Typhimurium ST313 D23580, and broadly classified into five groups (Fig. S1). All 63 genes are >200 nt, indicating that their essentiality calls are reliable. Identification of the chromosomal *cysS* gene as not required in *S. enterica* Typhimurium ST313 D23580 provides support for our inter-strain essentiality analysis approach: a second orthologous *cysS* gene is encoded by the pBT1 plasmid of D23580, and we have previously shown experimentally that the chromosomal *cysS* gene is dispensable for growth [30].

Genes belonging to group 3 are of particular interest as they represent 22 genes that are not required by the two African *Salmonella* strains D7795 and D23580, but are required by the GEC strain P125109 (Fig. S1). Pseudogenization, and the consequent loss of gene function, is linked to host range restriction for *Salmonella *, as observed for *S. enterica* Typhi [86] and for the switch from an enteric to an extra-intestinal lifestyle by African *S. enterica* Typhimurium [12, 87]. For the future, it will be important to determine experimentally whether the functions of these 22 genes are truly dispensable for *S *. *enterica* Enteritidis CEAC D7795 and *S. enterica* Typhimurium ST313 D23580.

### Identification of fitness genes of *S. enterica* Enteritidis P125109 and D7795 during growth in LB, NonSPI2 and InSPI2 *in vitro* conditions

To build upon our identification of required genes in P125109 and D7795, we studied *in vitro* fitness of the two *S. enterica* Enteritidis strains by analysing the transposon mutants recovered after two passages in three different growth media under laboratory conditions: LB, NonSPI2 and InSPI2. The fitness genes required for growth in each media were identified by the insertion index and essentiality analysis (Table S3).

**Fig. 3.**
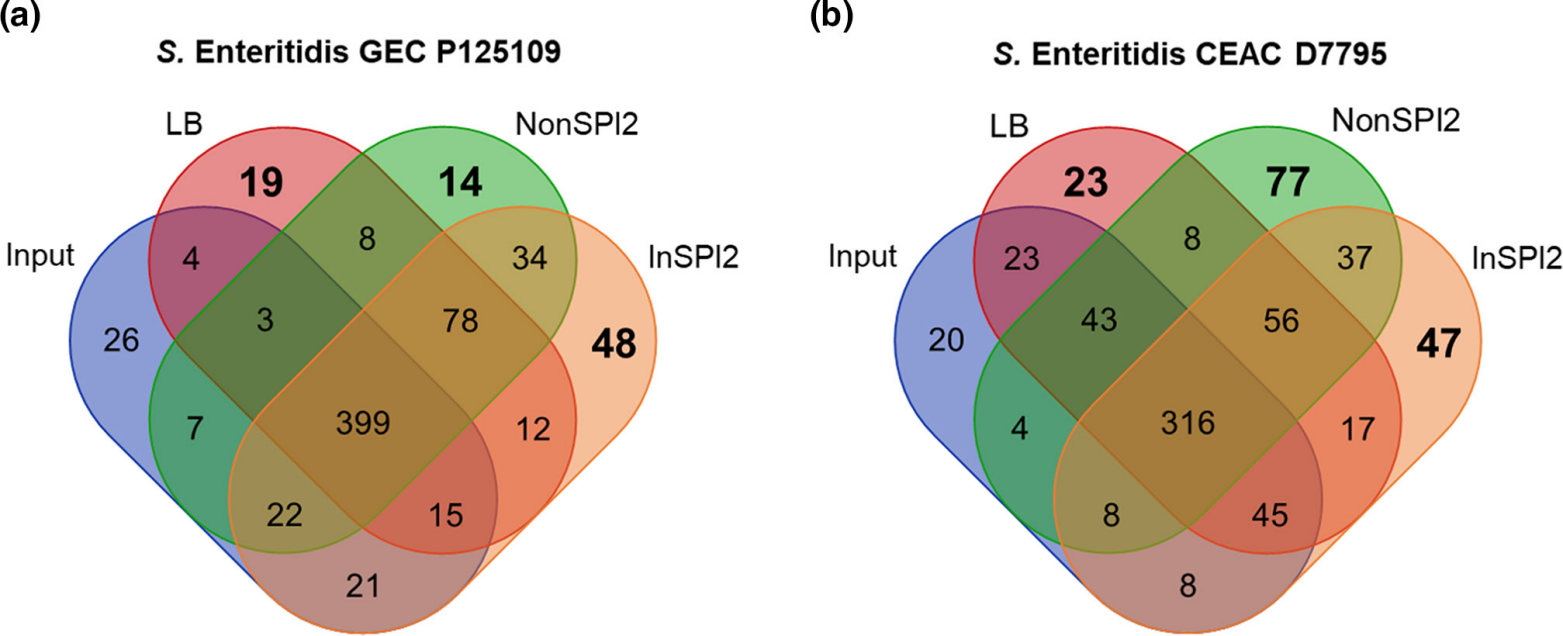
*
S *. *enterica* Enteritidis GEC P125109 and CEAC D7795 genes required for fitness in LB, NonSPI2 and InSPI2 media. The Venn diagrams compare the required genes in (a) P125109 or (b) D7795 with genes required for optimal growth in LB, NonSPI2 and InSPI2 media in the respective strains (Table S4). Image generated using http://bioinformatics.psb.ugent.be/webtools/Venn/.

Following two passages of the P125109 transposon library in complex LB media, 538 genes were designated as required, which included genes that were required in the input as well as genes that contributed to fitness for *in vitro* growth in LB: 531 genes in the chromosome and 7 genes in the pSENV plasmid. A total of 565 genes were required for optimal growth after two passages in NonSPI2 minimal media: 561 genes in the chromosome and 4 genes in the pSENV plasmid. Analysis of the InSPI2 output library identified 629 genes, with 622 genes located in the chromosome and 7 genes in the pSENV plasmid. The overlap between the required gene lists for Input, LB output, NonSPI2 output and InSPI2 output was determined to distinguish between the genes shared between all conditions and genes that were only required under specific conditions. We identified 19 genes for LB, 14 genes for NonSPI2 and 48 genes for InSPI2 that were required only in the specific media; these genes were categorized as LB-only, NonSPI2-only and InSPI2-only, respectively (Fig. 3a).

The P125109 LB-only required genes fall mainly into the major category of ‘information storage and processing’, with 7 out of 19 genes (37 %) belonging to this category, followed by 5 genes in the ‘metabolism’ major category (Fig. S2, Table S4). Compared to the NonSPI2-only and InSPI2-only fitness genes, fewer metabolism-related genes were required for optimal growth in LB, which reflects the nutrient-rich LB environment [88]. Among the 19 LB-only required genes, 6 genes (*xseB*, *cydA*, *recB*, *gidA*, *yjeA*, *arcA*) have been previously identified as required by *S *. *enterica* Typhi for growth in LB [46]. The *recB* gene, encoding an exonuclease subunit, was also reported to be required by *S. enterica* Typhimurium 14028 [50, 89] and *E. coli* [47] after several passages in LB. The identification of *recB* gene in multiple TIS screens highlights the importance of this gene for fitness in the LB environment.

The P125109 genes that were required for optimal growth in NonSPI2 and/or InSPI2 were mostly metabolism-related genes, confirming the nutritional deficiencies encountered by the bacteria grown in synthetic minimal media. Genes required for optimal growth in both NonSPI2 and InSPI2 included *pur* genes (for purine biosynthesis) and *aro* genes (for aromatic amino acid biosynthesis). The InSPI2-only required genes included several genes associated with SPIs and RODs; the *dksA* gene was also identified, which plays a key role in the stringent response and has been experimentally validated as required for growth of *S. enterica* Typhimurium in minimal medium [90, 91].

For CEAC strain D7795, 541 genes, 559 genes and 532 genes were designated as required in LB, NonSPI2 and InSPI2, respectively, following two passages in the growth media. After removing genes that had been identified as required in the input, there were 23 LB-only required genes, 77 NonSPI2-only required genes and 47 InSPI2-only required genes (Fig. 3b). As seen in P125109, most of the D7795 LB-only required genes were involved in either metabolism (12 out of 23 genes; 52%) or information storage and processing (6 out of 23 genes; 26 %) processes (Fig. S2, Table S4). Five genes identified as required in D7795 for LB growth had been reported to be required by *S *. *enterica* Typhimurium previously: *ppiB*, a peptidyl-prolyl isomerase; *cydA*, cytochrome oxidase d subunit; *crp*, cAMP-activated global transcriptional regulator [92]; *rstB*, the sensor kinase of the *rstAB* two-component system; and *rnfD*, a component of the electron transport chain [30].

Most of the D7795 genes identified as required for optimal growth in NonSPI2 and InSPI2 minimal media were, as expected, metabolism-related, including genes involved in amino acid transport and metabolism (E), nucleotide transport and metabolism (F), and energy production and conversion (C) (Table S4). Similar to P125109, the D7795 *dksA* gene was also identified as required for growth in InSPI2 media. Several SPI-1 and SPI-2 genes and a SPI-1 effector, SopE, were required for growth in NonSPI2, while two SPI-5 genes (*pipB* and *sORF26*) and one ROD9 gene (*SEN1001*) were required for growth in InSPI2.

There were 78 and 56 genes designated as required in all growth media (LB, NonSPI2 and InSPI2) for P125109 and D7795, respectively (Fig. 3, Table S4). These genes represent the biological processes required by P125109 and D7795 for growth in laboratory conditions, and include genes such as *ftsK*, *icdA*, *rpiA*, *yheN*, *rfa* and *atp*, which are also required by *S. enterica* Typhimurium ST19 14 028 during *in vitro* growth [89, 93].

Overall, we conclude that similar functional categories of genes were required by both P125109 and D7795 for optimal growth in LB, NonSPI2 and InSPI2 media. The functional categories were mainly involved in energy production and conversion (C), carbohydrate transport and metabolism (G), amino acid transport and metabolism (E), nucleotide transport and metabolism (F), and transcription (K) (Fig. S2). As with all global mutagenesis approaches, identified gene hits should be validated in the future by individual gene deletions followed by phenotypic characterization of the knockout mutants.

### *S. enterica* Enteritidis D7795 survives and replicates better in macrophages than *S. enterica* Enteritidis P125109

**Fig. 4.**
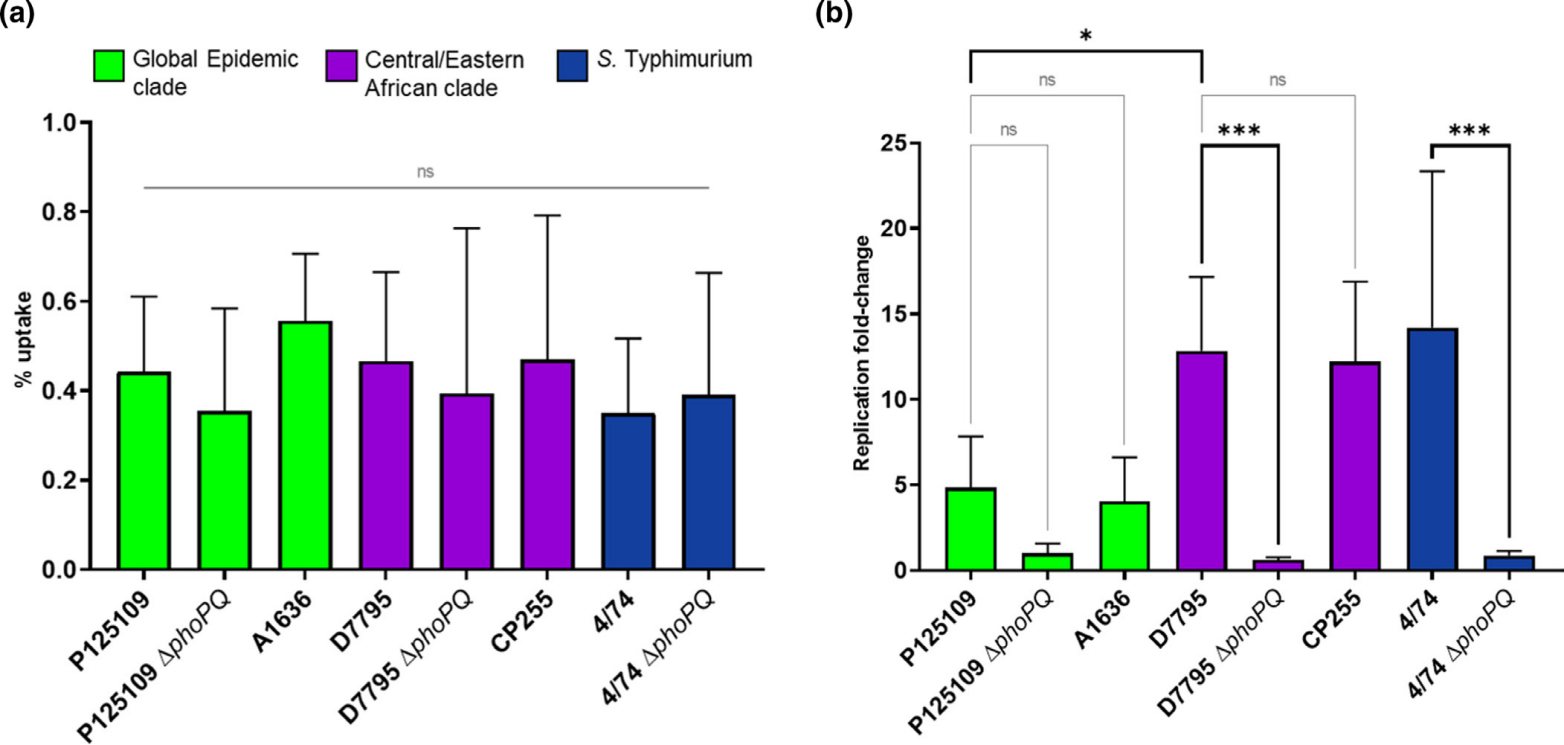
*
S *. *enterica* Enteritidis CEAC D7795 displays higher levels of intracellular survival and replication than *S. enterica* Enteritidis GEC P125109 in murine macrophages. *S. enterica* Enteritidis strains are colour-coded by clades, as defined by Feasey *et al.*[10]: green, GEC; purple, CEAC. The *S. enterica* Typhimurium 4/74 and 4/74 Δ*phoPQ* (blue) strains were included as additional controls. (a) Uptake of *Salmonella* strains by RAW 264.7 macrophages, shown as the percentage of the infecting inoculum recovered (c.f.u.) at 1.5 h post-infection (p.i.). (b) Intra-macrophage replication of *Salmonella *, shown as the ratio of intracellular bacteria (c.f.u.) recovered at 15.5 h p.i. compared to the c.f.u. recovered at 1.5 h p.i., as fold-change. Both panels (a and b) represent mean values obtained from five independent experiments with three replicates each, and error bars show standard deviation. The statistical tests used were one-way ANOVA, followed by Bonferroni’s multiple comparison test to compare selected pairs of means. ns, *P*≥0.05; *, *P*<0.05; ***, *P*<0.001.

To date, virulence phenotypes of CEAC D7795 have only been assessed in an avian infection model [10]. Survival and replication within macrophages is an important step in systemic *Salmonella* infections [53]. We compared the interaction of *S. enterica* Enteritidis GEC P125109 and CEAC D7795 with RAW 264.7 macrophages, together with two additional *S. enterica* Enteritidis strains, A1636 and CP255. A1636 is a GEC isolate from Africa, while CP255 belongs to the same CEAC as D7795 that originated from the Democratic Republic of Congo [10, 16]. Δ*phoPQ* deletion mutants were constructed in P125109 and D7795 by λ Red recombineering as negative controls for the infection studies. The PhoPQ two-component regulatory system is required for the survival of *S. enterica* Typhimurium within macrophages [94, 95], and a previous genome-wide screen of *S. enterica* Enteritidis P125109 Tn*5* mutants showed that *phoPQ* insertion mutants were negatively selected during murine infection [17]. The *S. enterica* Typhimurium ST19 strains 4/74 [96] and 4/74 Δ*phoPQ* [97] were also included for comparison. All *S. enterica* Enteritidis and *S. enterica* Typhimurium strains tested in this experiment were taken up by RAW 264.7 macrophages at similar levels. Importantly, we found that the CEAC isolates showed significantly higher levels of intra-macrophage replication than GEC isolates at 15.5 h post-infection (Fig. 4).

### Identification of fitness genes of *S. enterica* Enteritidis P125109 and D7795 following macrophage infection

Having established that the CEAC strain D7795 survives and replicates better in macrophages than GEC strain P125109, the respective transposon libraries were used to investigate the process of intracellular infection of RAW 264.7 macrophages. Each pool of transposon mutants was grown in LB (Input_LB) then passaged once through murine macrophages. Intra-macrophage bacteria were recovered at 12 h post-infection and grown in LB for 10 h to generate the output culture (Output_MAC). A fraction of the input was sub-cultured in LB for 10 h to ascertain the effect of growth in LB broth culture on the composition of the transposon library (Output_LB_10 h). Genomic DNA from the input and output samples was purified and prepared for Illumina sequencing of DNA adjacent to the transposon, as described in Methods.

Genes that modulated the intracellular survival and replication of *Salmonella* in RAW 264.7 macrophages were identified by comparing the macrophage output samples with the input samples and calculating the changes in frequency of reads mapped to each gene, expressed as log_2_(fold-change) (FC) [30]. A gene is considered to exhibit differential fitness if its log_2_FC value is less than 1 (attenuated fitness) or greater than 1 (increased fitness) with a *P* value <0.05. Genes affecting growth in LB for 10 h were similarly identified by comparing the LB output samples with the input samples. Required genes were identified from the input sample using the insertion index and excluded from the list of differential fitness genes.

**Fig. 5.**
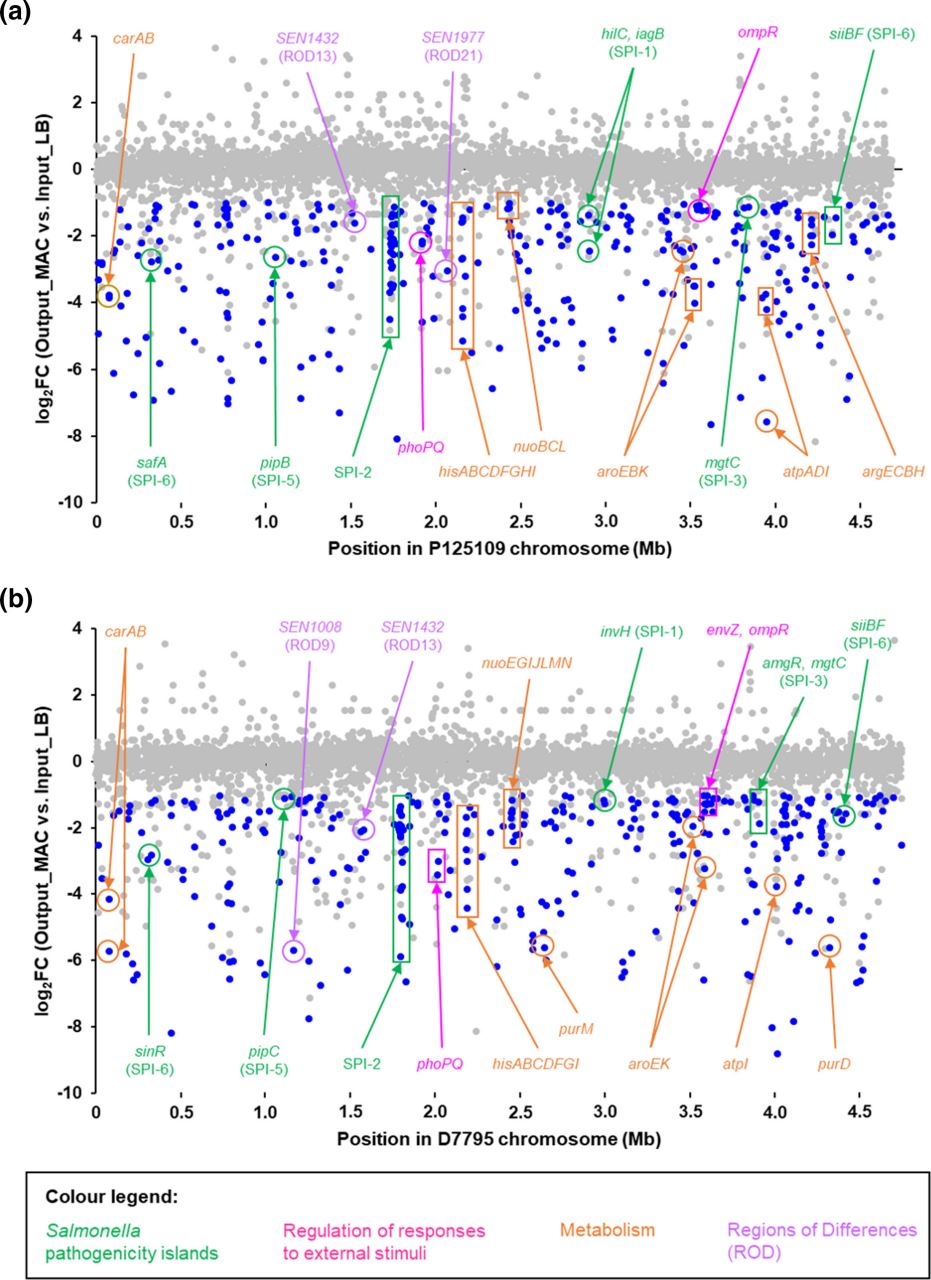
Macrophage-attenuated genes of *S. enterica* Enteritidis GEC P125109 and CEAC D7795. The *S. enterica* Enteritidis GEC P125109 (a) and CEAC D7795 (b) transposon libraries were used to infect RAW 264.7 macrophages for 12 h. Relative abundance of each mutant was determined by comparing the frequency of sequenced reads mapped to each gene after the infection (Output_MAC) to the initial inoculum (Input_LB). Each position on the *x*-axis represents the starting nucleotide position of each gene locus on the *Salmonella* chromosome, and the *y-*axis represents the log_2_ fold-change (FC) of changes in abundance of mapped reads. Loci with significant reduction in abundance (log_2_FC <−1, *P*<0.05) are shown in blue (attenuated fitness). Grey dots include both loci with significant increase in abundance (log_2_FC > 1, *P*<0.05) and loci with *P*≥0.05. DeSeq2 analysis of the TIS macrophage data is presented in Table S5.

Following 12 h macrophage infection, a total of 479 P125109 genes were identified as required from essentiality analysis of the input libraries. In total, transposon insertions in 327 genes were associated with attenuated replication within macrophages (Fig. 5a, Table S5). To identify genes important for fitness inside macrophages but not for growth in laboratory media, we first compared the 327 macrophage-attenuated genes with the 124 genes that showed attenuation in 10 h LB growth when disrupted by a transposon insertion (Table S5). This analysis identified 227 genes that only attenuated fitness of P125109 during macrophage infection, and not during 10 h growth in LB (Fig. S3, Table S4).

The resulting 227 genes were then cross-referenced with genes required for growth in the LB, NonSPI2 and InSPI2 *in vitro* laboratory conditions tested in this study (Fig. S3). We identified a total of 320 genes that were ‘macrophage-associated’. Of these, 177 genes were ‘macrophage-specific’, only having reduced fitness during macrophage infection with no impact upon growth *in vitro* (Fig. S3, Table S4). The terms macrophage-specific and macrophage-associated have been defined previously [30], and are explained in the legend to Fig. S3.

The 177 macrophage-specific P125109 genes included many well-characterized genes important for intra-macrophage survival of *S. enterica* Typhimurium, such as 22 SPI-2 genes, *mgtC* from SPI-3 and several global regulatory systems that control *Salmonella* virulence (e.g. *ompR*, *phoQ*, *ssrB*). Two SPI-1 genes, *hilC* and *iagB*, were also identified. There were 74 genes related to various metabolic processes, including arginine biosynthesis (*arg* genes), histidine biosynthesis (*his*) genes, the Tricarboxylic Acid Cycle (TCA) cycle (*sucD*) and oxidative phosphorylation (*ndh*), reflecting the nutritional stresses encountered by the bacteria within the macrophage environment. Three genes only present in P125109 were also identified as macrophage-specific, namely *SEN0912* (encoding a hypothetical protein) and two tRNA genes (*tRNA-Ala* and *tRNA-Thr*). No genes from the ROD9 region were identified, despite reports that ROD9-associated genes played a role in virulence in macrophage and animal infection [17, 21, 98]. Based on COG classifications, the majority of the 177 macrophage-specific genes were associated with nucleotide transport and metabolism (F, 17 %), amino acid transport and metabolism (E, 10 %), transcription (K, 8 %) and translation (J, 9 %).

Genes important for intra-macrophage fitness of D7795 were identified as described earlier. Essentiality analysis of the input sample identified 432 required genes, and these genes were excluded from the list of genes that caused differential fitness in macrophage infection. Transposon insertions in 329 genes caused attenuation (log_2_ fold-change < −1, *P*<0.05) during RAW macrophage infection (Fig. 5b). Of the 329 genes, 78 genes exhibited reduced fitness during growth in LB for 10 h. Cross-referencing the 251 genes with the genes required for *in vitro* growth under laboratory conditions identified a total of 325 macrophage-associated genes and 201 macrophage-specific genes (Fig. S3, Table S4). Similar to the findings for P125109, the 201 D7795 macrophage-specific genes are primarily located in the SPI regions. Specifically, most of the attenuated mutants were located in SPI-2; several others were identified in SPI-3 (*SEN3578*, *amgR*, *mgtC*), SPI-4 (*siiF*), SPI-5 (*pipC*), SPI-6 (*SEN0286*) and SPI-10 (*SEN4249*). Transposon insertions in *SEN1008* from ROD9 (SPI-19) also attenuated macrophage fitness. Distribution of the COG functional categories of the macrophage-specific genes is broadly similar to that in P125109, with nucleotide transport and metabolism (F) and amino acid transport and metabolism (E) genes forming the largest categories.

Transposon insertion in 61 P125109 and 18 D7795 genes resulted in increased fitness in macrophage infection (log_2_ fold-change >1, *P*<0.05), and include the *rfa*/*rfb* genes that are responsible for lipopolysaccharide (LPS) O-antigen biosynthesis (Table S5). Increased intra-macrophage fitness conferred by transposon disruption in the *rfa*/*rfb* genes was also observed in *S. enterica* Typhimurium infection of RAW 264.7 macrophages [30, 99]. The *rfb* genes are involved in O-antigen synthesis, while the *rfa* genes mediate LPS core synthesis [100]. As noted by Canals *et al.* [30], *Salmonella* mutants lacking the LPS O-antigen are phagocytosed at higher levels than wild-type strains by murine macrophages [101]. This likely accounts for the increased number of *S. enterica* Enteritidis *rfa*/*rfb* mutants recovered in the macrophage output, and reiterates the importance of interpreting the results of a mutant screen in the context of the experimental model used [92, 99].

### *S. enterica* Enteritidis P125109 and D7795 genes that modulate intra-macrophage fitness are required for virulence in other *Salmonella* serovars

The *S. enterica* Enteritidis GEC P125109 and CEAC D7795 genes associated with differential fitness in macrophage infection were compared with other infection models and *Salmonella* serovars. Genes affecting intra-macrophage fitness of *S. enterica* Typhimurium ST313 D23580 [30] were identified by re-processing and analysis of the data using pipelines described in Methods. Genes required for *Salmonella* infection in other serovars and/or infection models were retrieved from published data sets using parameters established by the authors of the original papers [17, 99, 102, 103]. Only orthologous genes were used in the comparisons.

**Fig. 6.**
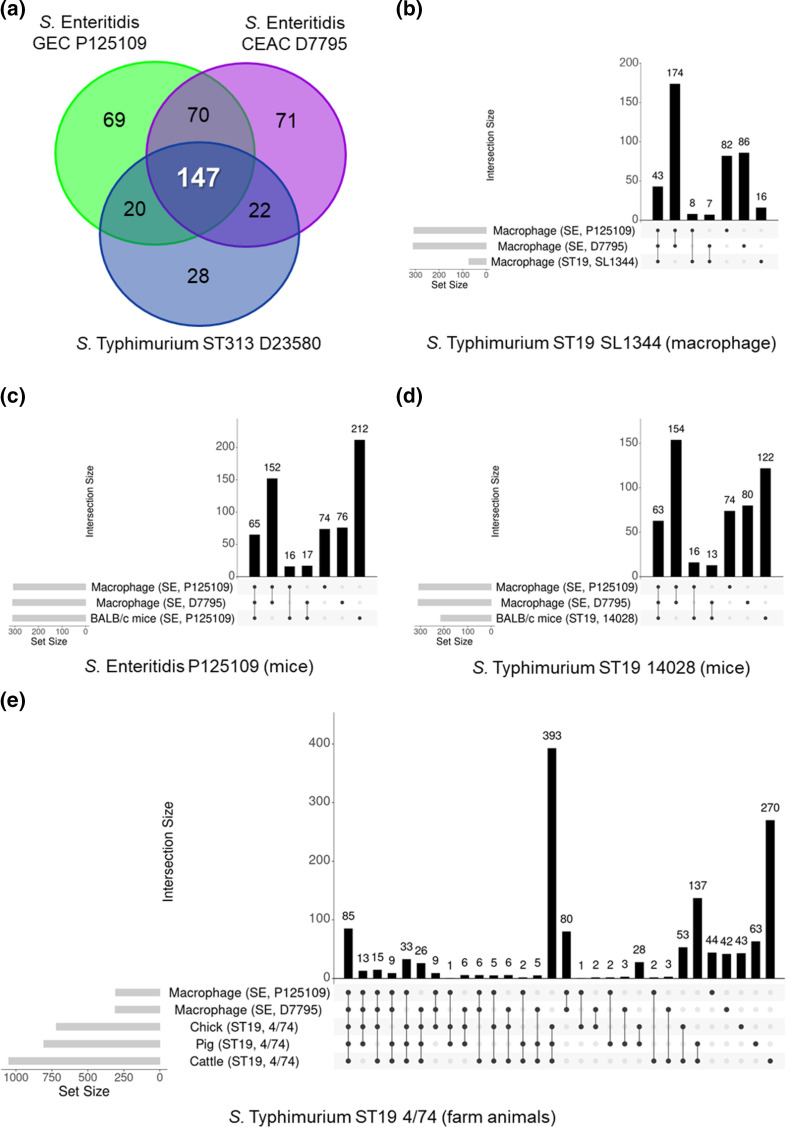
Genes that modulate intra-macrophage fitness of *S. enterica* Enteritidis GEC P125109 and CEAC D7795 are required for virulence in other infection models. Macrophage-attenuated genes in *S. enterica* Enteritidis GEC P125109 and CEAC D7795 were compared with genes associated with virulence for: (a) *S. enterica* Typhimurium ST313 D23580 [30] and (b) ST19 SL1344 [99] in macrophage infection; (c) P125109 [17] and (d) *S. enterica* Typhimurium ST19 14028 [102] in BALB/c mice infection; and (e) *S *. *enterica* Typhimurium ST19 4/74 in food-related animal infection models [103]. Only orthologous genes were included in the analyses (Table S6). UpSet plots were generated using Intervene [105].

We found that mutation of a total of 147 genes caused attenuated macrophage infection for *S. enterica* Enteritidis P125109, D7795 and *S. enterica* Typhimurium ST313 D23580 (Fig. 6a, Table S6). There was also significant overlap with genes required for *Salmonella* virulence in mice and other infection models, demonstrating that *S. enterica* Enteritidis shares many virulence genes in common with other serovars (Fig. 6b–e). Many of these genes include the well-characterized regulatory systems that control *Salmonella* virulence (the *phoPQ* two-component regulators and *ompR*, an element of the *ompR–envZ* two-component regulatory system), and SPI-2 genes that encode structural components of the type III secretion system, and genes involved in purine and aromatic amino acids biosynthesis (Table S6, Fig. S4).

### *S. enterica* Enteritidis D7795 and *S. enterica* Typhimurium ST313 D23580 do not share novel virulence factors

We used the TIS data to search for genes involved in intra-macrophage virulence that were only found in African *Salmonella* strains. Focusing on the three-way comparison between *S. enterica* Enteritidis GEC P125109, CEAC D7795 and *S. enterica* Typhimurium ST313 D23580, 22 genes were identified as modulating macrophage fitness in invasive African *Salmonella* strains D7795 and D23580 but not in gastroenteritis-associated P125109 (Fig. 6a). Cross-referencing these 22 genes with *Salmonella* virulence genes identified previously [17, 99, 102, 103] showed that all 22 genes have been implicated in at least one other infection model (Table S6). We conclude that no novel virulence factors that modulate intra-macrophage fitness are shared by the two African *Salmonella* strains.

### Candidate novel macrophage fitness genes that were unique to *S. enterica* Enteritidis P125109 or D7795

The three-way comparison between *S. enterica* Enteritidis GEC P125109, CEAC D7795 and *S. enterica* Typhimurium ST313 D23580 identified mutations in 69 P125109 genes and 71 D7795 genes that attenuated intra-macrophage fitness (Fig. 6a). We investigated the role of these 69 and 71 genes in other infection models or in *S. enterica* Typhimurium, and did a detailed comparison against genes identified in other published studies [17, 99, 102, 103]. We identified a total of 22 P125109 genes and 39 D7795 genes that were only associated with attenuated macrophage fitness in a single strain (Tables 1 and 2). These 22 and 39 genes represent candidate novel virulence genes for P125109 and D7795, respectively, and include strain-specific genes (i.e. genes without orthologues in the other *Salmonella* strains referenced in this study) (e.g. *SEN0912* in P125109 and three tRNA genes of D7795, locus tags D7795_02738, D7795_03122 and D7795_04774) and genes with orthologues in the other *Salmonella* strains and serovars that were not associated with attenuated fitness during macrophage infection (e.g. *lipB*::Tn*5*, *gatR*::Tn*5*, *hscB*::Tn*5*, *rnfE*::Tn*5* and *sopD2*::Tn*5* are attenuated in D7795 but not in P125109, *S. enterica* Typhimurium D23580 and/or LT2). Experimental validation of these candidates by individual gene deletion mutation will be necessary to verify a role in intra-macrophage replication.

**Table 1.** Candidate novel macrophage fitness genes in *S. enterica* Enteritidis P125109 P125109 ID, P125109 locus tags from re-annotation [16]; SEN ID, *S. enterica* Enteritidis identifiers [13]; D7795 ID, D7795 locus tags from re-annotation [16]; D23580 ID, *S. enterica* Typhimurium ST313 strain D23580 identifiers [31]; LT2 ID, *S. enterica* Typhimurium ST19 strain LT2 identifiers [106]; P125109 log_2_FC, log_2_FC (macrophage output vs input). All values have *P* values <0.05. FC, Fold-change.

Name	Product	P125109 ID	SEN ID	D7795 ID	D23580 ID	LT2 ID	P125109 log_2_FC
**No orthologue in D7795, D23580 nor LT2**							
*SEN0912*	Hypothetical protein	P125109_01026	SEN0912	Absent	Absent	Absent	−1.41
*tRNA-Ala*	tRNA-Ala(ggc)	P125109_02722	Absent	Absent	Absent	Absent	−2.28
*tRNA-Thr*	tRNA-Thr(ggt)	P125109_04470	Absent	Absent	Absent	Absent	−3.47
**Present in D7795, D23580 and/or LT2 but only attenuated in P125109**							
*tpke11*	None	P125109_00013	Absent	D7795_00014	STMMW_ncRNA_1	Absent	−3.32
*yabC*	Conserved hypothetical protein	P125109_00141	SEN0121	D7795_00143	STMMW_01261	STM0120	−1.23
*yaeD*	Conserved hypothetical protein	P125109_00284	SEN0256	D7795_00285	STMMW_02532	STM0248	−5.05
*SEN0328*	Hypothetical protein	P125109_00373	SEN0328	D7795_00374	STMMW_04151	STM0345	−2.14
*SEN0532*	Hypothetical protein	P125109_00595	SEN0532	D7795_00602	STMMW_06191	STM0551	−1.64
*sucD*	Succinyl-CoA synthetase alpha chain	P125109_00774	SEN0689	D7795_00799	STMMW_07961	STM0739	−1.35
*SEN0907*	Putative ion:amino acid symporter	P125109_01022	SEN0907	D7795_01047	STMMW_10141	STM1003	−1.01
*yedD*	Putative lipoprotein	P125109_01175	SEN1044	D7795_01257	STMMW_19441	STM1964	−3.78
*cheZ*	Chemotaxis protein CheZ	P125109_01224	SEN1088	D7795_01307	STMMW_18981	STM1915	−1.81
*lrhA*	NADH dehydrogenase operon transcriptional regulator	P125109_02623	SEN2312	D7795_02672	STMMW_23521	STM2330	−1.57
*iagB*	Cell invasion protein	P125109_03091	SEN2719	D7795_03189	STMMW_28391	STM2877	−2.45
*ygbK*	Conserved hypothetical protein	P125109_03136	SEN2756	D7795_03234	STMMW_28801	STM2917	−1.59
*sORF75*	None	P125109_03192	Absent	D7795_03290	STMMW_29261	Absent	−1.75
*yggT*	Putative membrane protein	P125109_03353	SEN2944	D7795_03449	STMMW_30621	STM3101	−1.50
*glpR*	Glycerol-3-phosphate regulon repressor	P125109_03805	SEN3348	D7795_03904	STMMW_35121	STM3523	−1.25
*prlC*	Oligopeptidase A	P125109_03882	SEN3417	D7795_03981	STMMW_35831	STM3594	−1.28
*wecG*	Probable UDP-N-acetyl-d-mannosaminuronic acid transferase	P125109_04235	SEN3734	D7795_04333	STMMW_39041	STM3929	−1.26
*serB*	Putative phosphoserine phosphatase	P125109_04914	SEN4334	D7795_05011	STMMW_45211	STM4578	−1.38
*yjjY*	Conserved hypothetical protein	P125109_04937	SEN4355	D7795_05034	STMMW_45421	STM4599	−2.03

**Table 2.** Candidate novel macrophage fitness genes in *S. enterica* Enteritidis D7795 D7795 ID, D7795 locus tags from re-annotation [16]; SEN ID, *S. enterica* Enteritidis identifiers [13]; P125109 ID, P125109 locus tags from re-annotation [16]; D23580 ID, *S. enterica* Typhimurium ST313 strain D23580 identifiers [31]; LT2 ID, *S. enterica* Typhimurium ST19 strain LT2 identifiers [106]; D7795 log_2_FC, log_2_FC (macrophage output vs input). All values have *P* values <0.05. FC, Fold-change.

Name	Product	D7795 ID	SEN ID	P125109 ID	D23580 ID	LT2 ID	D7795 log_2_FC
**No orthologue in P125109, D23580 nor LT2**							
*group_972*	Tail fibre assembly protein	D7795_02043	Absent	Absent	Absent	Absent	−1.33
*tRNA-Arg*	tRNA-Arg(cct)	D7795_02738	Absent	Absent	Absent	Absent	−1.64
*tRNA-Arg*	tRNA-Arg(acg)	D7795_03122	Absent	Absent	Absent	Absent	−3.08
*tRNA-Gly*	tRNA-Gly(gcc)	D7795_04774	Absent	Absent	Absent	Absent	−5.59
*group_1609*	Hypothetical protein, partial	D7795_05141	Absent	Absent	Absent	Absent	−1.28
**Present in P121509, D23580 and/or LT2 but only attenuated in D7795**							
*yacC*	Conserved hypothetical protein	D7795_00194	SEN0172	P125109_00193	STMMW_01731	STM0167	−1.33
*SEN0286*	Transposase (fragment)	D7795_00327	SEN0286	P125109_00326	STMMW_03133	Absent	−1.24
*queA*	*S*-Adenosylmethionine:tRNA ribosyltransferase-isomerase	D7795_00442	SEN0387	P125109_00436	STMMW_04741	STM0404	−1.26
*tgt*	Queuine tRNA-ribosyltransferase tRNA-guanine transglycosylase	D7795_00443	SEN0388	P125109_00437	STMMW_04751	STM0405	−1.64
*lipB*	Lipoate-protein ligase B (lipoate biosynthesis protein B)	D7795_00703	SEN0604	P125109_00677	STMMW_07001	STM0635	−4.97
*phoL*	PhoH-like ATP-binding protein	D7795_00738	SEN0638	P125109_00713	STMMW_07341	STM0669	−1.52
*nagC*	*N*-Acetylglucosamine repressor	D7795_00752	SEN0646	P125109_00727	STMMW_07401	STM0682	−1.02
*sopD2*	Putative sopD2 type III secretion system effector protein	D7795_01014	SEN0876	P125109_00989	STMMW_09831	STM0972	−2.06
*pipC*	Cell invasion protein	D7795_01153	SEN0954	P125109_01070	STMMW_11021	STM1090	−1.12
*fliD*	Flagellar hook associated protein (FliD)	D7795_01261	SEN1048	P125109_01179	STMMW_19401	STM1960	−1.27
*fliC*	Flagellin	D7795_01262	SEN1049	P125109_01180	STMMW_19381	STM1959	−1.17
*motB*	Motility protein B	D7795_01300	SEN1081	P125109_01217	STMMW_19051	STM1922	−1.19
*flhA*	Flagellar biosynthesis protein FlhA	D7795_01310	SEN1090	P125109_01227	STMMW_18961	STM1913	−1.14
*rnfE*	Putative electron transport complex protein rnfE	D7795_01882	SEN1593	P125109_01796	STMMW_14571	STM1454	−1.89
*ydiV*	Conserved hypothetical protein	D7795_01999	SEN1700	P125109_01912	STMMW_13511	STM1344	−1.24
*SEN2359*	Conserved hypothetical protein	D7795_02722	SEN2359	P125109_02673	STMMW_23991	STM2377	−1.03
*hscB*	Co-chaperone protein hscB	D7795_02906	SEN2520	P125109_02858	STMMW_25571	STM2540	−1.98
*pphB*	Possible serine/threonine protein phosphatase	D7795_03219	SEN2745	P125109_03121	STMMW_28701	STM2907	−1.28
*icc*	Conserved hypothetical protein	D7795_03541	SEN3026	P125109_03444	STMMW_31431	STM3183	−1.27
*gatR*	Galactitol utilization operon repressor	D7795_03624	SEN3097	P125109_03525	STMMW_32621	STM3262	−1.16
*SraG*	None	D7795_03646	Absent	P125109_03547	STMMW_ncRNA_221	Absent	−1.57
*greA*	Transcription elongation factor	D7795_03664	SEN3132	P125109_03565	STMMW_32981	STM3299	−1.18
*damX*	DamX protein	D7795_03865	SEN3311	P125109_03766	STMMW_34751	STM3485	−1.29
*mtlR*	Mannitol operon repressor (mannitol repressor protein)	D7795_04081	SEN3509	P125109_03982	STMMW_36751	STM3687	−1.18
*asnA*	Asparagine synthetase A	D7795_04282	SEN3691	P125109_04183	STMMW_38611	STM3877	−2.93
*yneB*	Putative aldolase	D7795_04494	SEN3868	P125109_04397	STMMW_40431	STM4078	−1.13
*yjaG*	Conserved hypothetical protein	D7795_04594	SEN3955	P125109_04498	STMMW_41221	STM4169	−1.06
*dgkA*	Diacylglycerol kinase	D7795_04646	SEN4005	P125109_04550	STMMW_41861	STM4236	−1.53
*yjfI*	Conserved hypothetical protein	D7795_04792	SEN4136	P125109_04696	STMMW_43141	STM4370	−1.59
*yjfO*	Putative exported protein	D7795_04801	SEN4145	P125109_04705	STMMW_43231	STM4379	−1.24
*pepA*	Cytosol aminopeptidase	D7795_04888	SEN4230	P125109_04792	STMMW_44231	STM4477	−1.03
*repA2*	CDO50996.1	D7795_05036	SEN_p0018	P125109_04939	SLT-BT0121	PSLT023	−1.97
*pefB*	CDO50994.1	D7795_05038	SEN_p0016	P125109_04941	SLT-BT0111	PSLT019	−1.30
*traY*	CDO51040.1	D7795_05058	SEN_p0063	P125109_04972	SLT-BT0801	PSLT076	−1.82

### Perspective

Since the identification of genetic variants of *S. enterica* Typhimurium and *S. enterica* Enteritidis that are highly associated with bloodstream infections in sub-Saharan Africa, only the *S. enterica* Typhimurium pathovariant has been an active focus of research [104]. Functional genomic studies involving African *S. enterica* Enteritidis have lagged behind. Here, we used a global mutagenesis approach to compare gene function between bloodstream infection-associated *S. enterica* Enteritidis CEAC strain D7795 and gastroenteritis-associated GEC strain P125109. Our findings reveal broad similarities between the gene sets required for growth under laboratory conditions and macrophage infection by P125109 and D7795. The majority of these genes were also important for fitness in other *Salmonella* serovars and infection models. We identified 39 genes that could encode candidate novel virulence factors for *S. enterica* Enteritidis CEAC strain D7795, and are worthy of further investigation.

## Supplementary Data

Supplementary material 1
