# Supplementary material for: Genome-wide fitness analysis identifies genes required for in vitro growth and macrophage infection by African and global epidemic pathovariants of Salmonella enterica Enteritidis
Source: Microb Genom. 2023 May 23;9(5):mgen001017. doi: 10.1099/mgen.0.001017 (PMC10272866; doi:10.1099/mgen.0.001017)
Supplement: Supplementary material 1 [file mgen-9-1017-s001.pdf]

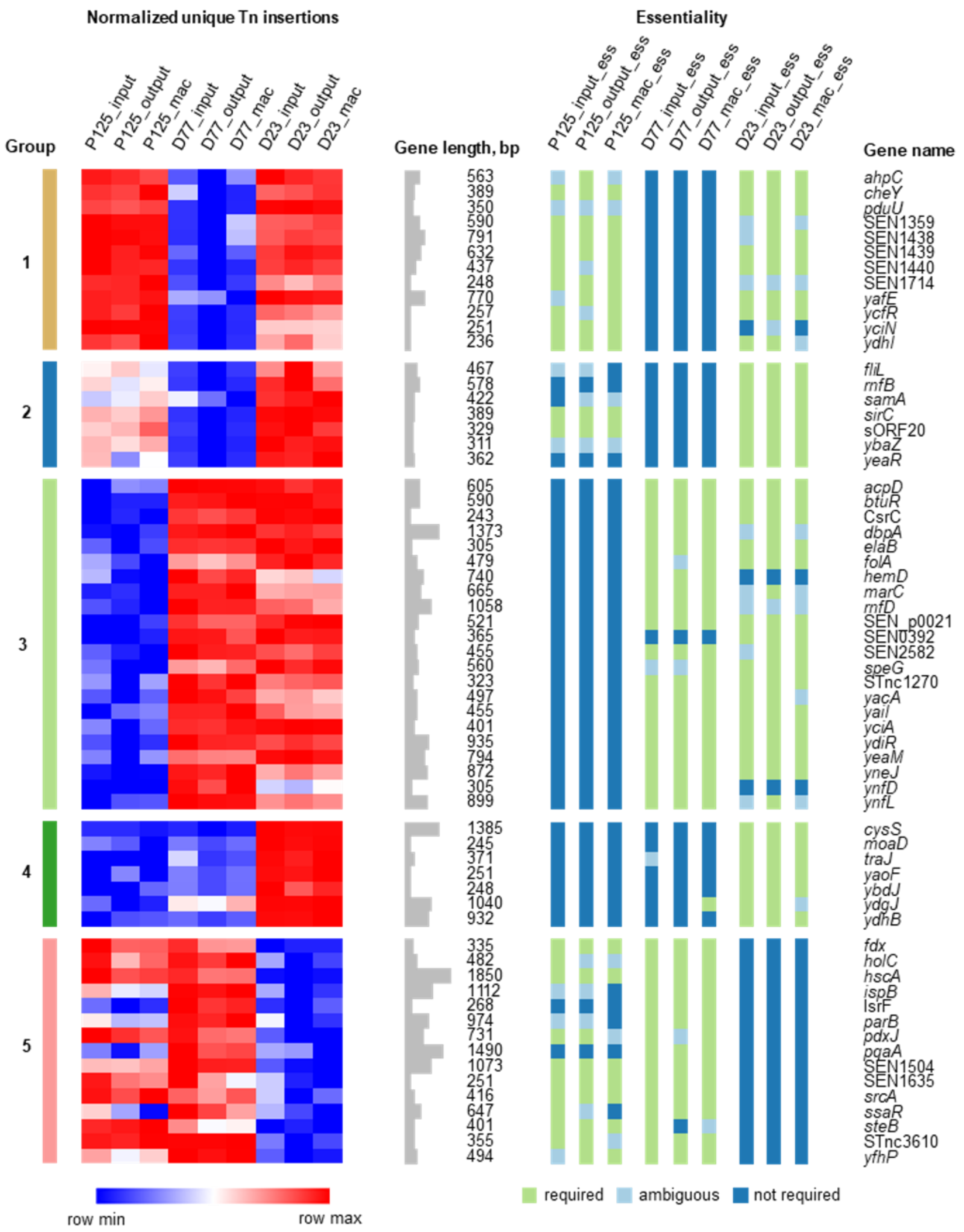

Fig S1. Inter-strain essentiality analysis identifies 63 genes that are differentially required between *S.* Enteritidis P125109, *S.* Enteritidis D7795 and *S.* Typhimurium D23580

Transposon insertion read counts are represented as a heat map, with red indicating many insertions and blue indicating very few insertions. Essentiality calls (based on insertion indices) from LB input, LB output and macrophage output libraries are represented by green and blue squares. Samples included: P125_input (P125109 LB input), P125109_output (P125109 LB output), P125_mac (P125109 macrophage output), D77_input (D7795 LB input), D77_output (D7795 LB output), D77 mac (D7795 macrophage output), D23_input (D23580 LB input), D23_output (D23580 LB output) and D23_mac (D23580 macrophage output).

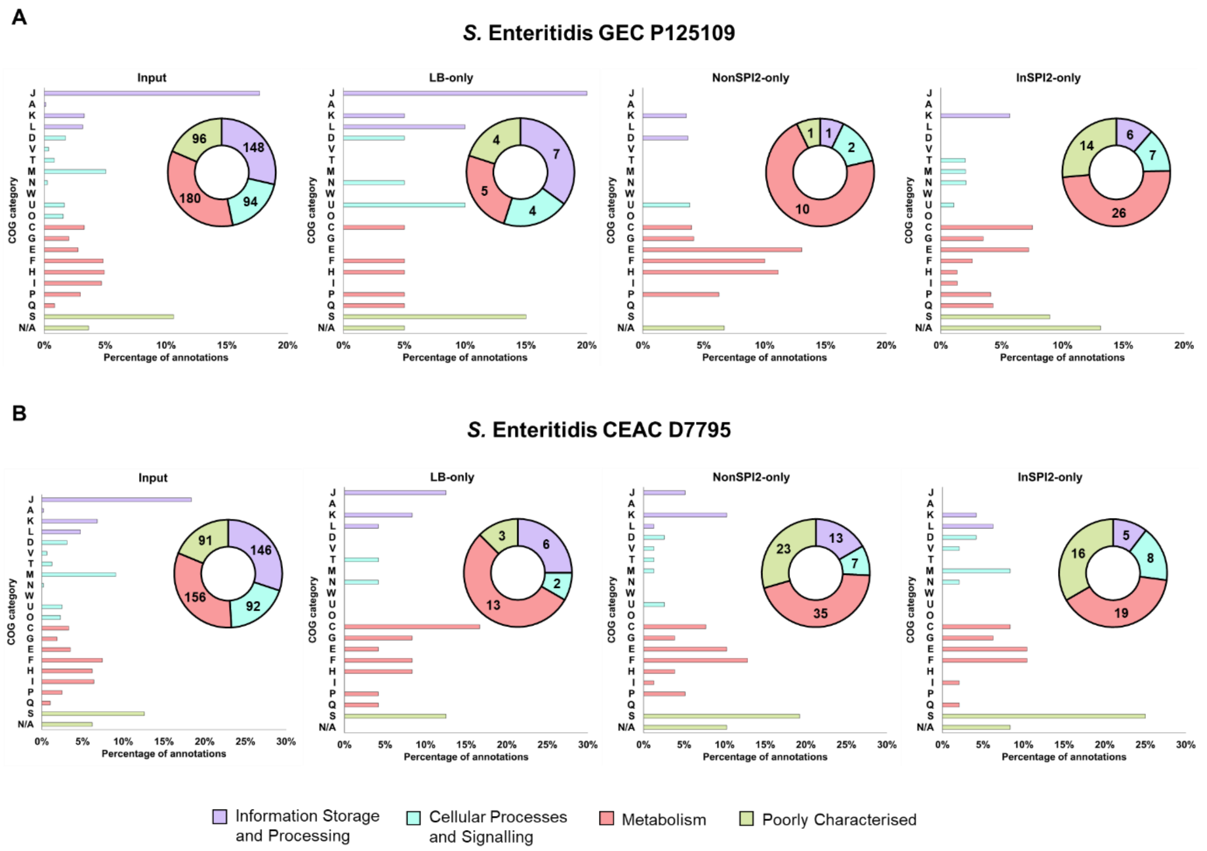

Fig S2. Distribution of Cluster of Orthologous Genes (COG) annotations in genes required by *S.* Enteritidis P125109 and D7795 for optimal growth in LB, NonSPI2 and InSPI2

Doughnut charts (insets) show the distribution of COG annotations in the four major functional categories (Information Storage and Processing, Cellular Processes and Signalling, Metabolism, Poorly Characterised), with total numerical counts in each major category shown. “Input” bar chart shows COG annotations from all genes identified as required in the respective *S.* Enteritidis Input libraries (497 genes for P125109 and 467 genes for D7795), whereas the “LB-only”, “NonSPI2-only” and “InSPI2-only” bar charts considered only the genes specific to that growth media (Fig 3). COG categories: J, Translation, ribosomal structure and biogenesis; A, RNA processing and modification; K, Transcription; L, Replication, recombination and repair; D, Cell cycle control, cell division, chromosome partitioning; V, Defense mechanisms; T, Signal transduction mechanisms; M, Cell wall/membrane/envelope biogenesis; N, Cell motility; W, Extracellular structures; U, Intracellular trafficking, secretion, and vesicular transport; O, Posttranslational modification, protein turnover, chaperones; C, Energy production and conversion; G, Carbohydrate transport and metabolism; E, Amino acid transport and metabolism; F, Nucleotide transport and metabolism; H, Coenzyme transport and metabolism, I, Lipid transport and metabolism; P, Inorganic ion transport and metabolism; Q, Secondary metabolites biosynthesis, transport and catabolism; S, Function unknown; N/A, not assigned.

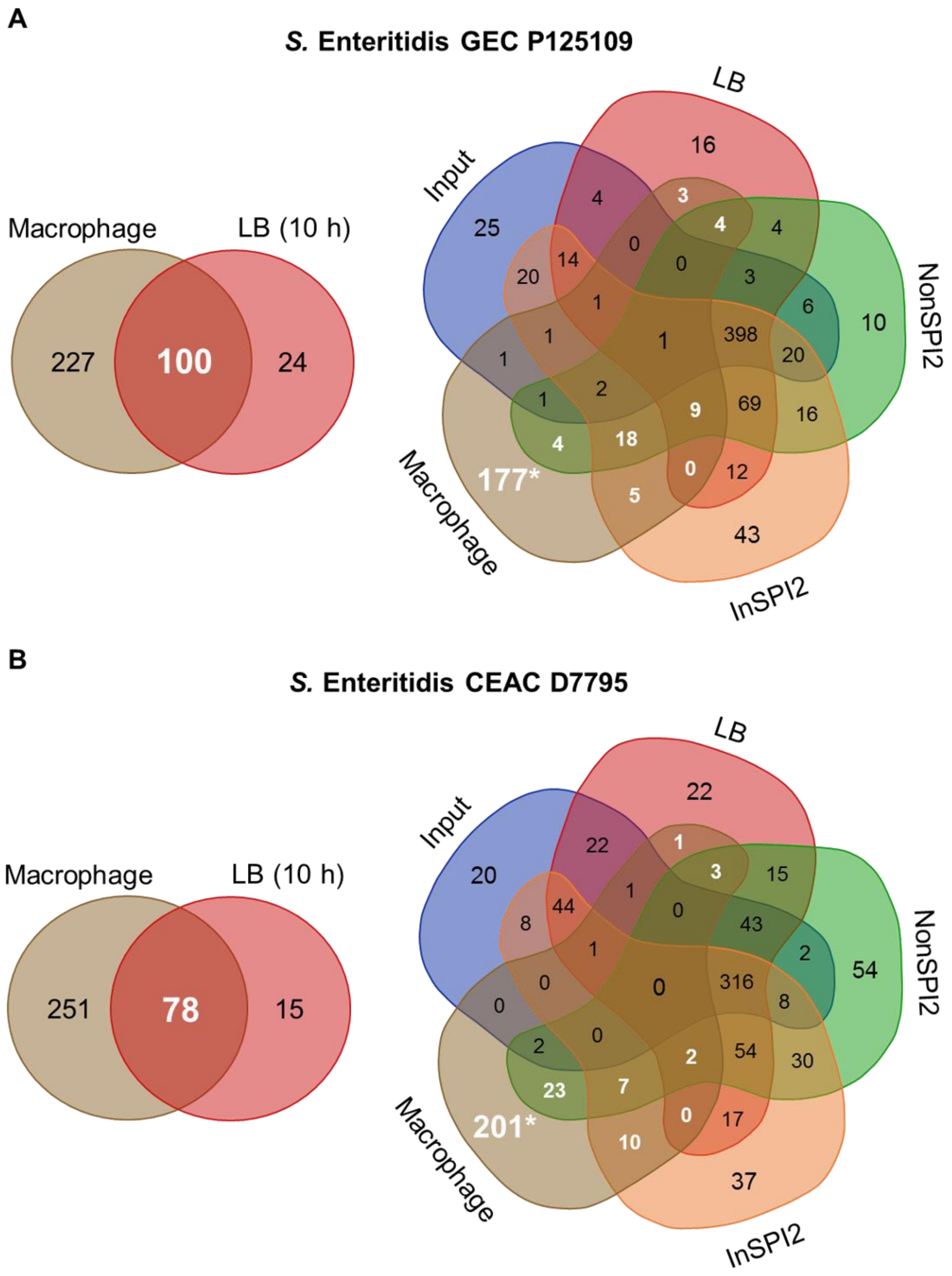

Fig S3. Macrophage-specific and macrophage-associated genes in *S.* Enteritidis P125109 and D7795

Genes that modulated the intracellular survival and replication of (A) *S.* Enteritidis P125109 and (B) D7795 in RAW 264.7 macrophages (identified by $\log_2$FC [Output_MAC vs. Input_LB] < −1 and *P*-value < 0.05) were compared with genes affecting growth in LB for 10 h (two-way Venn diagram in both panels A and B). The resulting 227 and 251 macrophage-attenuated fitness mutants in P125109 and D7795, respectively, were then compared with the genes required for *in vitro* growth under laboratory conditions (five-way Venn diagram). The numbers highlighted in white represent the "macrophage-associated" genes, and the numbers highlighted with an asterisk (*) represent the "macrophage-specific" genes. Venn diagrams were generated using http://bioinformatics.psb.ugent.be/webtools/Venn/.

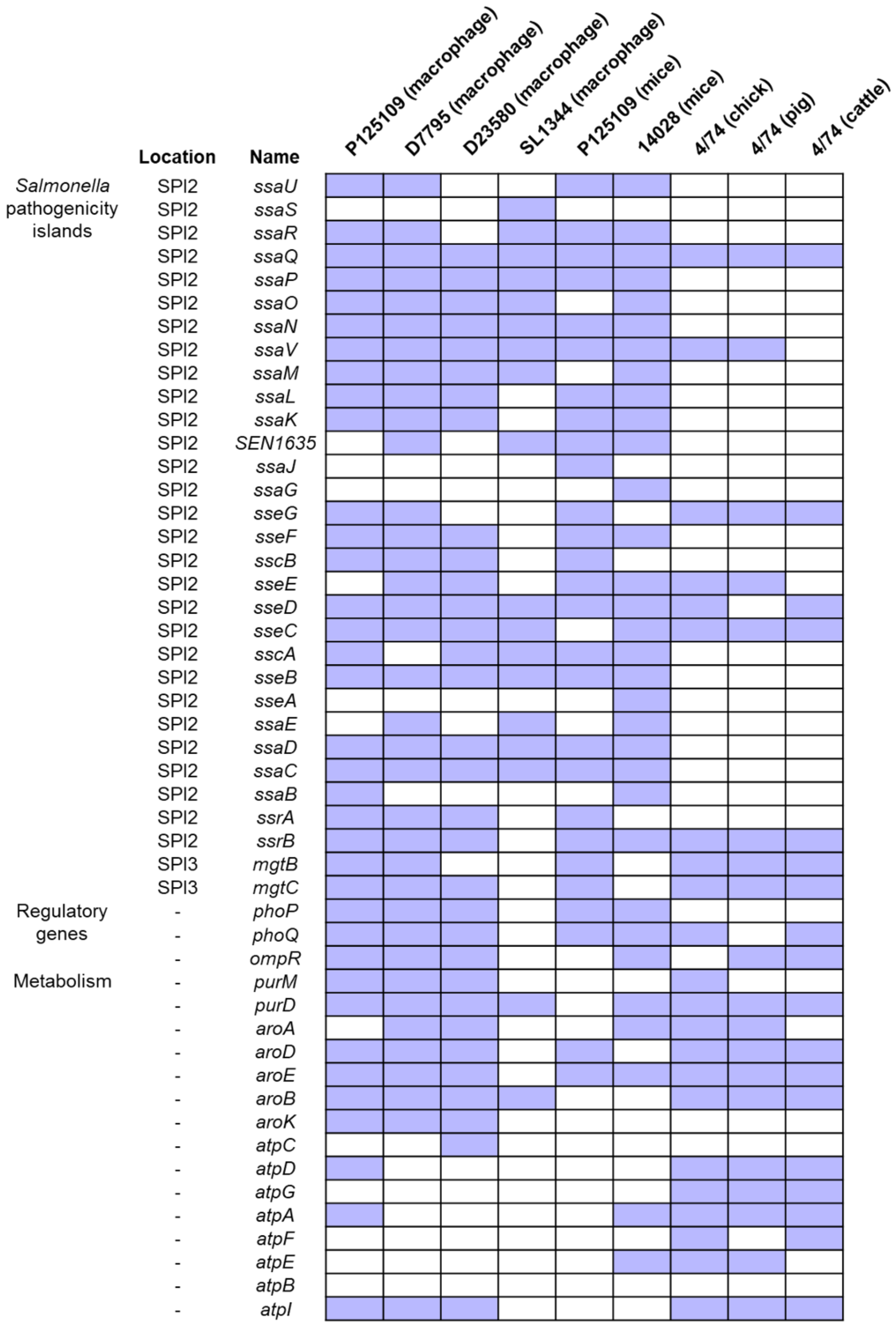

Fig S4. Macrophage-fitness genes in of *S.* Enteritidis P125109 and D7795 with reported roles in other *Salmonella* infection models

The figure shows 49 genes that are required for macrophage fitness in *S.* Enteritidis P125109 and/or D7795 as identified in this study, and their reported roles in other *Salmonella* infection models. Blue box indicates that the gene is involved in *Salmonella* fitness in the specified strain and infection model. D23580 (macrophage) = *S.* Typhimurium ST313 D23580 in macrophage infection [1]; SL1344 (macrophage) = *S.* Typhimurium ST19 SL1344 in macrophage infection [2]; P125109 (mice) = *S.* Enteritidis P125109 in BALB/c mice infection [3]; 14028 (mice) = *S.* Typhimurium ST19 14028 in BALB/c mice infection [4]; and 4/74 (chick), 4/74 (pig), 4/74 (cattle) = *S*. Typhimurium ST19 4/74 in food-related animal infection models [5].

References

1. **Canals R, Chaudhuri RR, Steiner RE, Owen S V., Quinones-Olvera N, *et al.*** The fitness landscape of the African *Salmonella* Typhimurium ST313 strain D23580 reveals unique properties of the pBT1 plasmid. *PLoS Pathog* 2019;15:e1007948.
2. **Venturini E, Svensson SL, Maaß S, Gelhausen R, Eggenhofer F, *et al.*** A global data-driven census of *Salmonella* small proteins and their potential functions in bacterial virulence. *microLife* 2020;1:1–20.
3. **Silva CA, Blondel CJ, Quezada CP, Porwollik S, Andrews-Polymenis HL, *et al.*** Infection of mice by *Salmonella enterica* serovar Enteritidis involves additional genes that are absent in the genome of serovar Typhimurium. *Infect Immun* 2012;80:839–849.
4. **Silva-Valenzuela CA, Molina-Quiroz RC, Desai P, Valenzuela C, Porwollik S, *et al.*** Analysis of two complementary single-gene deletion mutant libraries of *Salmonella* Typhimurium in intraperitoneal infection of BALB/c mice. *Front Microbiol* 2016;6:1455.
5. **Chaudhuri RR, Morgan E, Peters SE, Pleasance SJ, Hudson DL, *et al.*** Comprehensive assignment of roles for *Salmonella* Typhimurium genes in intestinal colonization of food-producing animals. *PLoS Genet* 2013;9:e1003456.
